# Music Therapy Versus Cognitive Behavioral Therapy via Telehealth for Anxiety in Survivors of Cancer: A Randomized Clinical Trial

**DOI:** 10.1200/JCO-25-00726

**Published:** 2026-01-06

**Authors:** Kevin T. Liou, Joke Bradt, M. Beatriz Currier, Raymond Baser, Katherine Panageas, Jodi MacLeod, Desiree Walker, Susan Q. Li, Ana Maria Lopez, Kelly McConnell, Jun J. Mao

**Affiliations:** ^1^ Integrative Medicine Service, Department of Medicine, Memorial Sloan Kettering Cancer Center, New York, NY; ^2^ Department of Creative Arts Therapies, College of Nursing and Health Professions, Drexel University, Philadelphia, PA; ^3^ Psychosocial Oncology, Miami Cancer Institute, Miami, FL; ^4^ Department of Epidemiology & Biostatistics, Memorial Sloan Kettering Cancer Center, New York, NY; ^5^ Society for Integrative Oncology, Pepper Pike, OH; ^6^ Young Survival Coalition, New York, NY; ^7^ Department of Medical Oncology, Thomas Jefferson University, Philadelphia, PA; ^8^ Department of Psychiatry and Behavioral Sciences, Memorial Sloan Kettering Cancer Center, New York, NY

## Abstract

**PURPOSE:**

Anxiety is prevalent, disruptive, and undertreated among survivors of cancer. Cognitive behavioral therapy (CBT) is the first-line treatment, but not all individuals have access, respond to treatment, or prefer this option because of stigma. Music therapy is effective for short-term anxiety reduction, but it is unknown whether it is noninferior to first-line CBT for long-term anxiety reduction.

**METHODS:**

This comparative effectiveness trial randomly assigned English- or Spanish-speaking survivors of cancer to seven weekly telehealth sessions of music therapy or CBT. The coprimary end points were changes in the Hospital Anxiety and Depression Scale (HADS) anxiety score at weeks 8 and 26. The noninferiority margin was 0.35 standard deviations, informed by a minimal clinically important difference (MCID) of 1.7 points. Secondary outcomes included fatigue, depression, insomnia, pain, cognitive dysfunction, and health-related quality of life.

**RESULTS:**

Among N = 300 patients, 74.7% was female, 76.5% was White, and 19.0% was Hispanic. At week 8, the mean change in HADS anxiety score was –3.12 (95% CI, –3.59 to –2.65) in music therapy and –2.97 (95% CI, –3.45 to –2.50) in CBT; the between-group difference was –0.15 (95% CI, –0.78 to 0.49), within the noninferiority margin of 1.20 (*P* < .001). At week 26, the mean change was –3.31 (95% CI, –3.78 to –2.85) in music therapy and –3.00 (95% CI, –3.47 to –2.53) in CBT; the between-group difference was –0.31 (95% CI, –0.95 to 0.32), within the noninferiority margin of 1.28 (*P* < .001). Both groups produced anxiety reductions exceeding the MCID and showed similar improvements in secondary outcomes.

**CONCLUSION:**

Music therapy is noninferior to CBT for anxiety in survivors of cancer. Both telehealth interventions produced clinically meaningful, durable improvements in anxiety.

## INTRODUCTION

Anxiety affects nearly one in two survivors of cancer^1,2^ and is associated with worse fatigue, other comorbid symptoms,^3,4^ increased health care expenditures,^5^ and negative quality of life.^6-8^ Although medications are available,^9^ polypharmacy is a growing concern in survivors of cancer.^10-13^ The ASCO guideline recommends cognitive behavioral therapy (CBT) in various delivery formats as the first-line treatment for anxiety.^14^ However, some patients are unable to complete a full treatment course^15,16^ or achieve clinically meaningful improvements.^17,18^ Individuals may also avoid CBT because of stigma associated with psychotherapy.^19-21^ A shortage of CBT providers presents additional barriers to access,^22,23^ highlighting the need for other effective anxiety treatments.

**CONTEXT**

**Key Objective**
Is music therapy as effective as cognitive behavioral therapy (CBT) for anxiety symptoms in survivors of cancer?
**Knowledge Generated**
In this randomized clinical trial that included 300 English- or Spanish-speaking survivors of cancer with anxiety symptoms, music therapy was noninferior to CBT for anxiety reduction at the end of treatment and 18 months post-treatment when delivered via telehealth. Both treatments produced clinically meaningful, durable improvements in anxiety with few adverse events.
**Relevance *(C. Zimmermann)***
Music therapy delivered via telehealth by board-certified music therapists may be considered in place of telehealth-delivered CBT as a treatment option for anxiety symptoms in survivors of cancer.*
*Relevance section written by *JCO* Associate Editor Camilla Zimmermann, MD, PhD, FRCPC.

Music therapy is an evidence-based intervention, in which board-certified music therapists guide patients through tailored music experiences to achieve therapeutic goals.^24^ This modality is available at approximately 50% of National Cancer Institute–designated cancer centers and community hospitals in the United States.^25,26^ In a Cochrane meta-analysis, music therapy was associated with reductions in anxiety and fatigue relative to usual care, with larger effect sizes observed for anxiety symptoms.^27^ Music has been shown to affect brain regions responsible for mood regulation and arousal, providing biological plausibility for these observed effects of music therapy on anxiety and comorbid symptoms, such as fatigue.^28,29^ The ASCO-Society for Integrative Oncology joint guideline recommends music therapy for anxiety.^30^ This growing evidence base presents a compelling rationale for considering music therapy as a treatment option alongside other first-line therapies for anxiety. However, most research of music therapy in oncology has only evaluated short-term anxiety reduction in hospitalized patients with patients cancer undergoing active treatments and has lacked long-term follow-up and active comparators. Therefore, its long-term comparative effectiveness relative to other first-line therapies for anxiety during cancer survivorship is unknown.

The pandemic accelerated telehealth adoption of music therapy and CBT, with recent estimates indicating that 46%-88% of providers offer telehealth services.^31,32^ Substantial research has demonstrated that telehealth CBT is as effective as in-person CBT for anxiety and other mental health conditions.^33-37^ However, few studies have compared telehealth CBT with other telehealth interventions.^38,39^ Research on delivering music therapy via telehealth is still in its infancy.^40-42^ To address these gaps, we conducted the Music Therapy versus Cognitive Behavioral Therapy for Cancer-related Anxiety (MELODY) study. The primary aim was to determine the comparative effectiveness of music therapy and CBT delivered via telehealth for anxiety in survivors of cancer. The primary hypothesis was that music therapy would be noninferior to first-line CBT for anxiety; a secondary hypothesis was that music therapy would be superior to CBT for fatigue symptoms co-occurring with anxiety.

## METHODS

### Trial Design

This two-arm, parallel-group randomized trial was conducted at the Memorial Sloan Kettering Cancer Center and the Baptist Health Miami Cancer Institute. The trial protocol (Supplementary Material) was published previously.^43^ The institutional review board at the Memorial Sloan Kettering Cancer Center approved the trial and served as the institutional review board of record. All participants provided informed consent.

### Population

The trial enrolled English- or Spanish-speaking adult survivors of cancer with elevated anxiety symptoms lasting at least 1 month. Patients were considered survivors of cancer if they had a previous cancer diagnosis of any type or stage, had completed active treatment, and had stable oncologic disease or no evidence of oncologic disease, as indicated in the medical chart. Patients were considered to have elevated anxiety symptoms if they scored ≥8 on the anxiety subscale of the Hospital Anxiety and Depression Scale (HADS). Participants were excluded if they completed active cancer treatment <1 month before enrollment; however, maintenance therapies were allowed. Participants were also excluded if they had self-reported or documented active suicidal ideation, bipolar disorder, schizophrenia, or substance use disorder or if they scored ≥10 on the Blessed Orientation-Memory-Concentration instrument indicating cognitive impairment. Since study interventions consisted of seven sessions, participants were ineligible if they had self-reported or documented history of receiving seven or more music therapy or CBT sessions for anxiety within the past 6 months. Access to Zoom videoconferencing was required to participate in the telehealth interventions.

### Recruitment and Random Assignment

Potential participants were identified by patient databases, clinician referrals, and self-referrals, and research staff reviewed their medical charts to determine cancer status. If basic eligibility criteria were met, patients were scheduled for a telehealth visit with a clinician who administered the HADS anxiety subscale and confirmed all eligibility criteria. The clinician also asked patients whether they had a diagnosis of generalized anxiety disorder or were taking medications for anxiety. Consecutively eligible patients were consented, enrolled, and asked to complete baseline assessments before random assignment.

Participants were randomly assigned evenly to music therapy or CBT, using a secure system with full allocation concealment and using randomly permuted blocks of random length, stratified by study site, current anxiety medication use (yes/no), and preferred language (English/Spanish). Therapists, patients, and research coordinators were aware of group assignment; however, the principal investigator and biostatistician were masked.

### Treatments

Music therapy was delivered on Zoom by four board-certified music therapists. The treatment protocol was informed by the social-cognitive processing model of emotional adjustment to cancer,^44,45^ which posits that having safe outlets for processing difficult experiences within the context of supportive social relationships is key to long-term anxiety reduction. The initial sessions used receptive music activities (eg, music-guided relaxation) to provide short-term anxiety reduction. Subsequent sessions progressed to collaborative songwriting,^46,47^ a music therapy approach designed to target the social-cognitive factors contributing to long-term anxiety reduction.^44,45^ The collaborative nature of songwriting, including brainstorming themes, drafting lyrics, and composing melodies, served to provide creative outlets for processing difficult experiences within a supportive therapeutic relationship. Participants completed homework to reinforce skills and serve as transitions to subsequent sessions.

CBT was delivered on Zoom by nine licensed social workers and one clinical psychologist. The treatment protocol was based on the cognitive behavioral model of anxiety and designed to address thoughts and behaviors that trigger or exacerbate anxiety.^48-50^ Session activities included psychoeducation, relaxation techniques, cognitive restructuring, and strategies for planning activity engagement and managing worries.^51,52^ Participants received a workbook and completed homework that reinforced skills and concepts from the sessions.

The frequency, duration, and number of sessions mirrored the treatment schedules of mental health interventions described in the literature and real-world practice.^53,54^ Interventionists received group supervision from a board-certified music therapist or a licensed clinical psychologist. Sessions were recorded on secure, encrypted servers and reviewed using treatment fidelity checklists outlining the core intervention components. All sessions of the first three patients for each therapist were reviewed, then two randomly selected sessions per patient for the next three patients, and one randomly selected session every fifth patient to check for protocol drift. Sessions were delivered with fidelity if at least 80% of checklist items were completed; therapists were retrained if they did not deliver a session with fidelity.

### Outcomes

All outcomes were administered remotely using the Research Electronic Data Capture platform. The primary outcome was anxiety symptom severity assessed using the HADS anxiety subscale. Its reliability, validity, and factor structure are established in cancer populations.^55,56^ Previous research identified a within-individual minimal clinically important difference (MCID) of 1.7 points.^57^ The HADS anxiety subscale was administered at baseline and weeks 4, 8, 16, and 26; the score changes from baseline to weeks 8 and 26 were the coprimary end points.

As secondary outcomes, we administered the Brief Fatigue Inventory,^58^ HADS depression subscale,^55,56^ Insomnia Severity Index,^59^ Brief Pain Inventory, Functional Assessment of Cancer Therapy—Cognitive Function version 3,^60^ and Patient-Reported Outcomes Measurement Information System—Global Health, at baseline and weeks 4, 8, 16, and 26.

We tracked anxiety medication use using 1-week medication diaries, which were completed remotely by participants at baseline, week 8, and week 26. We also administered a validated treatment expectancy scale at baseline.^61^

### Statistical Analysis

We designed the trial to assess whether music therapy is noninferior to CBT based on changes in the HADS anxiety subscale score at weeks 8 and 26. Our sample size was selected to provide an 80% power for testing the noninferiority hypotheses, one at week 8 and one at week 26, while maintaining our overall Type I error at 0.05 for these two primary end point comparisons. The assumptions in the power and sample size calculations were identical for the two tests. Each test assumed 15% attrition and was based on a two-sample t-test of differences between the arms in their change scores with a one-sided significance threshold of 0.025 (controlling the overall Type I error at 0.05 for the two primary end point comparisons). The change scores were assumed to be distributed as standard normal variables with a standard deviation (SD) of 1.0. Given these assumptions, randomly assigning 300 participants in a 1:1 ratio would achieve an 80% power to detect noninferiority of music therapy to CBT at either time point within a noninferiority margin of D = 0.35 × SD on the HADS anxiety subscale score. Based on an expected SD of 4.2 from previous research,^62^ this margin translates to an expected D = 0.35 × 4.2 = 1.47, which is smaller than the established within-individual MCID of 1.7 points for the HADS anxiety subscale. In the absence of an established between-group MCID for the HADS anxiety subscale, we used the within-individual MCID to inform the noninferiority margin. Establishing noninferiority would suggest that differences between music therapy and CBT are not clinically meaningful. The actual SD values of the HADS anxiety subscale at weeks 8 and 26 were 3.42 and 3.65, respectively; thus, the noninferiority margins were 1.20 and 1.28.

Following the intention-to-treat principle, we analyzed outcomes using linear mixed-effects models, suitable for repeated continuous measures. This approach accounts for within-individual correlations, estimates between-group differences, and includes participants with missing data.^63^ The models included the treatment arm, the time point (categorical), baseline stratification variables (anxiety medication use, preferred language, study site), the time-by-arm interaction, and a participant-specific random intercept. The models constrained the treatment arms to have a common baseline mean, reflecting the prerandomization timing of the baseline assessment.^64^

For the primary outcome, we tested noninferiority at weeks 8 and 26 by evaluating time-by-arm interaction coefficients from the linear mixed-effects model for HADS anxiety subscale scores to determine whether music therapy was noninferior to CBT. We also defined treatment response rates as the proportion of participants with a reduction of ≥1.7 points (MCID of the HADS anxiety subscale) from baseline. Treatment response rates were calculated at weeks 8 and 26 and compared between arms using chi-square tests. We also performed sensitivity analyses to include only participants with generalized anxiety disorder. Only one participant experienced cancer recurrence, so we did not perform sensitivity analyses excluding those with recurrence.

Secondary outcomes, including fatigue, were analyzed using similar linear mixed-effects models to evaluate the change in scores over time, but differences between arms were evaluated using a superiority rather than noninferiority framework. The secondary hypothesis was that, compared with CBT, music therapy would be associated with significantly greater improvement in fatigue, as measured by the Brief Fatigue Inventory, at weeks 8 and 26 at the *P* < .05 significance threshold. We did not have prespecified secondary hypotheses about the other secondary outcomes, and we did not have a prespecified plan to adjust for multiple statistical tests for fatigue or the other secondary outcomes. In the presented results for secondary outcomes, the widths of the 95% CI for between-group differences have not been adjusted for multiplicity; therefore, these intervals should not be used to draw definitive conclusions about treatment effects.

We prespecified six variables to evaluate potential heterogeneity of treatment effects: sex, race, ethnicity, education, treatment expectancy, and time since cancer diagnosis. We evaluated these variables in separate models by expanding our linear mixed model to include the three-way interaction (and lower-order two-way interactions) between time, arm, and the prespecified variables. The presence of significant heterogeneity of treatment effect was tested using the Wald multivariate test for the three-way interaction. We adjusted the *P* values using the method of Benjamini and Hochberg^65^ to control the false discovery rate at α = .05. All statistical analyses were performed using R software, version 4.4.0.^66^

## RESULTS

### Population

**FIG 1.**
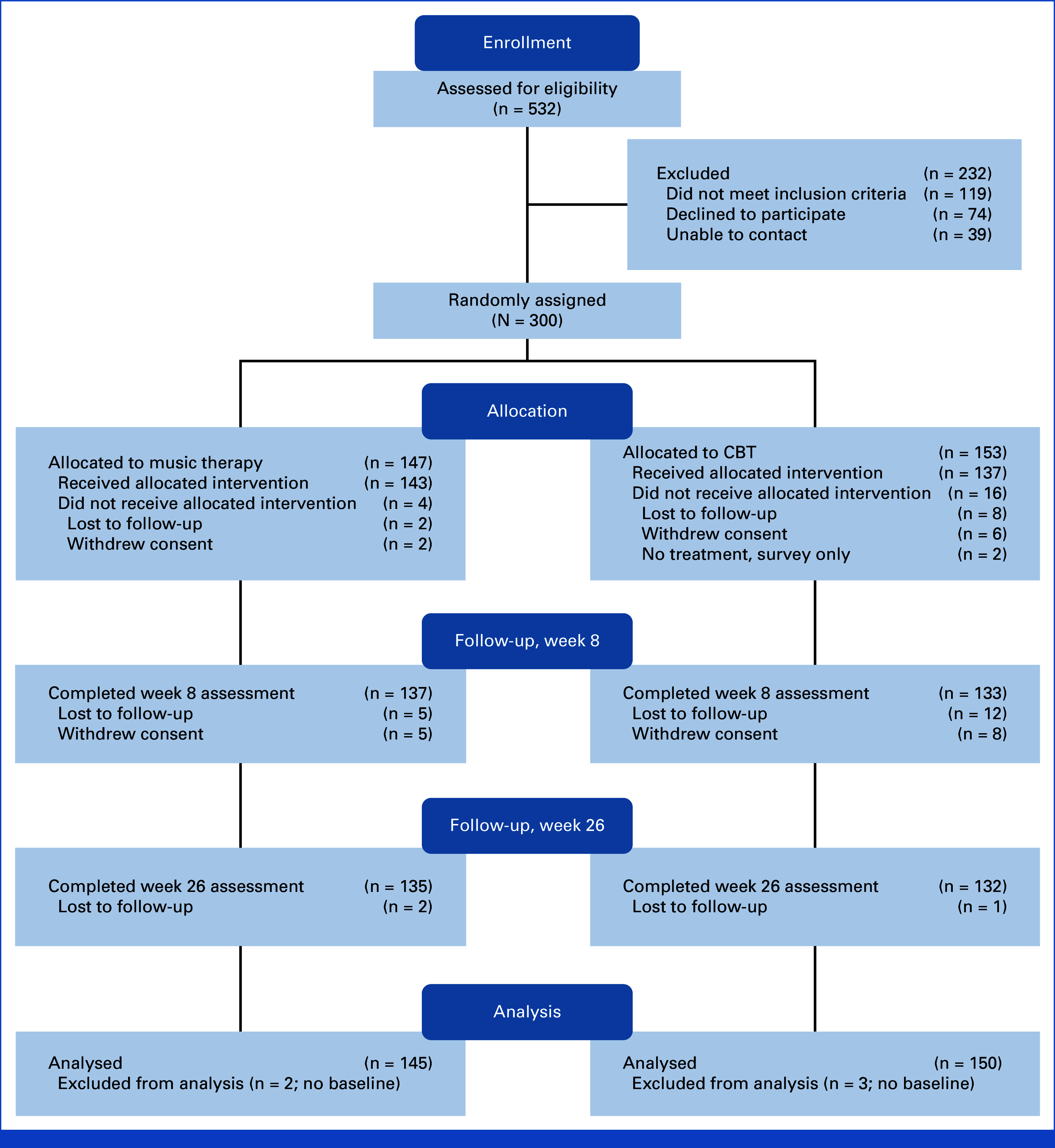
CONSORT diagram. This figure depicts participant flow through the MELODY trial. CBT, cognitive behavioral therapy; MELODY, Music Therapy versus Cognitive Behavioral Therapy for Cancer-related Anxiety.

From February 2022 to December 2023, we enrolled 300 participants (Fig 1). The mean age was 56.9 (SD, 13.2) years, 224 (74.7%) were female, 228 (76.5%) were White, and 57 (19%) were Hispanic. The baseline characteristics were similar between groups (Table 1).

**TABLE 1.** Characteristics of Study Participants

Characteristic	MT (n = 147)	CBT (n = 153)
Age, years (SD)	57.2 (13.5)	56.7 (13.0)
Female sex, No. (%)	108 (73.5)	116 (75.8)
White race, No. (%)	108 (74.0)	120 (79.0)
Hispanic ethnicity, No. (%)	23 (15.7)	34 (22.4)
Education level, No. (%)		
College or less	69 (47.6)	77 (52.4)
Graduate degree or more	76 (52.4)	70 (47.6)
Cancer type, No. (%)		
Breast	66 (44.9)	70 (45.8)
Prostate	12 (8.2)	10 (6.5)
Hematologic	22 (15.0)	25 (16.3)
GI	12 (8.2)	11 (7.2)
Other	35 (23.8)	37 (24.2)
Cancer stage, No. (%)		
0 to I	76 (53.1)	79 (53.4)
II to III	55 (38.5)	54 (36.5)
IV	10 (7.0)	13 (8.8)
Unresectable	2 (1.4)	2 (1.4)
Time since diagnosis, years (SD)	5.1 (6.9)	3.7 (5.4)
Cancer treatment history, No. (%)		
Surgery	105 (71.4)	107 (70.0)
Chemotherapy	66 (44.9)	76 (49.7)
Radiation therapy	67 (45.6)	76 (49.7)
Immunotherapy	14 (9.5)	14 (9.2)
Bone marrow transplant	2 (1.4)	5 (3.3)
Hormonal therapy	45 (30.6)	45 (29.4)
Coexisting anxiety disorders, No. (%)		
Generalized anxiety disorder	47 (32.0)	51 (33.3)
Panic disorder	3 (2.0)	6 (4.0)
Taking anxiety medication, No. (%)^a^	41 (33.3)	38 (32.5)
SSRI	26 (17.7)	25 (16.3)
SNRI	7 (4.8)	10 (6.5)
Benzodiazepine	32 (21.8)	32 (20.9)
Study site, No. (%)		
Memorial Sloan Kettering	125 (85.0)	128 (83.7)
Miami Cancer Institute	22 (15.0)	25 (16.3)

NOTE. Sociodemographics, anxiety disorders, and anxiety medication use were self-reported by participants. Cancer type, stage, and treatment history, as well as time since cancer diagnosis, were abstracted from the medical chart.

Abbreviations: CBT, cognitive behavioral therapy; MT, music therapy; SNRI, serotonin-norepinephrine reuptake inhibitor; SSRI, selective serotonin reuptake inhibitor.

^a^ Among 240 participants who provided medication diary data.

### Primary Outcome

**FIG 2.**
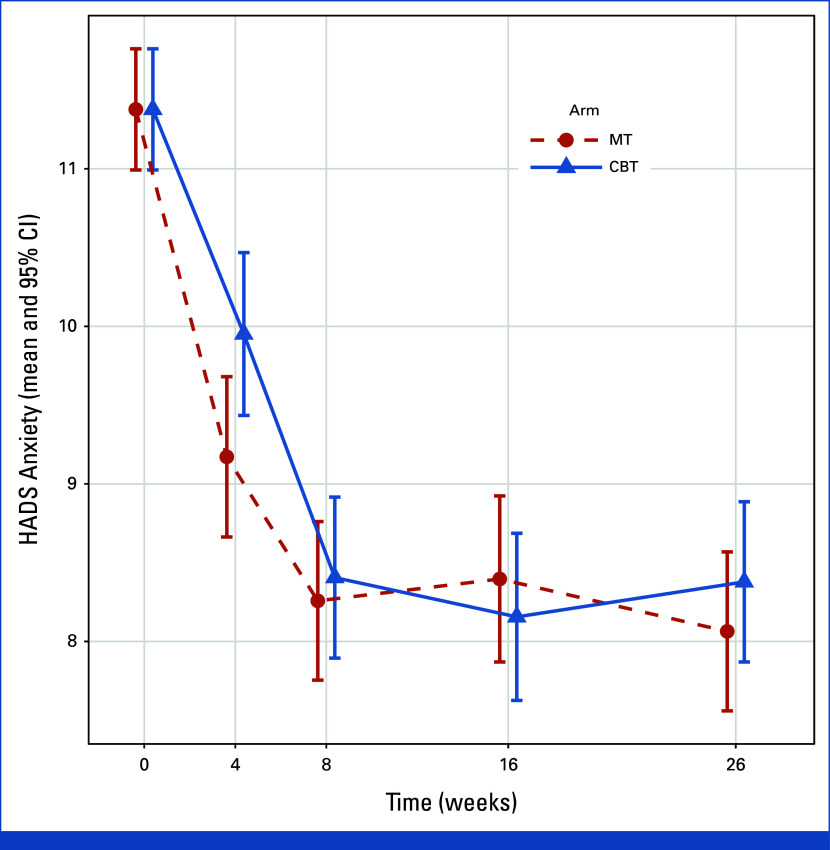
Mean HADS anxiety subscale score by treatment group over time. This figure shows the mean HADS anxiety subscale score by treatment group from baseline through the 26-week follow-up period of the study. CBT, cognitive behavioral therapy; HADS, Hospital Anxiety and Depression Scale; MT, music therapy.

**FIG A1.**
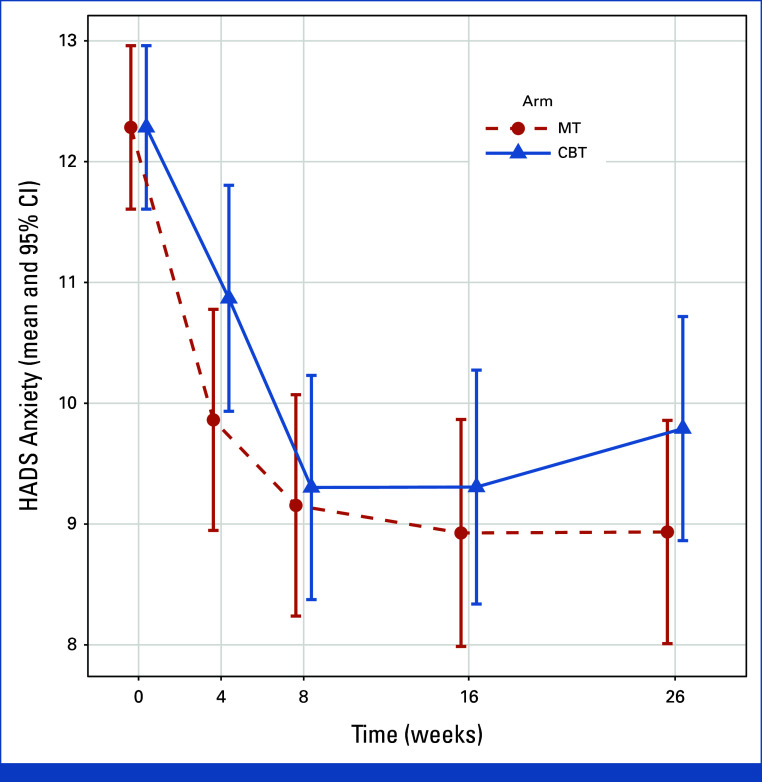
Sensitivity analyses of primary outcome including only patients with generalized anxiety disorder. This figure shows the mean HADS anxiety subscale score by treatment group from baseline through week 26 among patients with generalized anxiety disorder. CBT, cognitive behavioral therapy; HADS, Hospital Anxiety and Depression Scale; MT, music therapy.

The mean change in the HADS anxiety subscale score at week 8 was –3.12 (95% CI, –3.59 to –2.65) in music therapy and –2.97 (95% CI, –3.45 to –2.50) in CBT (between-group difference, –0.15 [95% CI, –0.78 to 0.49]; *P* < .001 for noninferiority). The mean change in the HADS anxiety subscale score at week 26 was –3.31 (95% CI, –3.78 to –2.85) in music therapy and –3.00 (95% CI, –3.47 to –2.53) in CBT (between-group difference, –0.31 [95% CI, –0.95 to 0.32]; *P* < .001 for noninferiority; Fig 2 and Table 2). Treatment response rates were similar between music therapy and CBT at week 8 (73.5% *v* 71.5%, *P* = .7) and week 26 (72.6% and 67.4%, *P* = .4). In the sensitivity analysis of patients with generalized anxiety disorder, the results were consistent with those of the primary analysis (Appendix Table A1 and Fig A1, online only).

**TABLE 2.** Primary and Secondary Outcomes

Outcome	MT (n = 145)	CBT (n = 150)	Between-Group Difference^a^
HADS anxiety subscale (primary)	Mean change (95% CI)	Mean change (95% CI)	Mean difference (one-sided 97.5% CI)
Week 8	–3.12 (–3.59 to –2.65)	–2.97 (–3.45 to –2.50)	–0.15 (∞ to 0.49)^b^
Week 26	–3.31 (–3.78 to –2.85)	–3.00 (–3.47 to –2.53)	–0.31 (∞ to 0.32)^c^
Brief fatigue inventory	Mean change (95% CI)	Mean change (95% CI)	Mean difference (95% CI)
Week 8	–1.18 (–1.50 to –0.85)	–1.36 (–1.69 to –1.02)	0.18 (–0.26 to 0.62)
Week 26	–1.02 (–1.35 to –0.69)	–1.31 (–1.65 to –0.97)	0.29 (–0.16 to 0.74)
HADS depression subscale	Mean change (95% CI)	Mean change (95% CI)	Mean difference (95% CI)
Week 8	–2.26 (–2.71 to –1.82)	–2.29 (–2.75 to –1.83)	0.03 (–0.59 to 0.64)
Week 26	–1.98 (–2.43 to –1.53)	–2.18 (–2.64 to –1.73)	0.20 (–0.41 to 0.81)
Insomnia severity index	Mean change (95% CI)	Mean change (95% CI)	Mean difference (95% CI)
Week 8	–3.07 (–3.85 to –2.30)	–2.82 (–3.61 to –2.03)	–0.25 (–1.31 to 0.80)
Week 26	–3.34 (–4.13 to –2.55)	–3.18 (–3.99 to –2.38)	–0.16 (–1.23 to 0.92)
Brief pain inventory—Severity	Mean change (95% CI)	Mean change (95% CI)	Mean difference (95% CI)
Week 8	–0.41 (–0.66 to –0.16)	–0.50 (–0.75 to –0.24)	0.09 (–0.25 to 0.44)
Week 26	–0.56 (–0.81 to –0.30)	–0.54 (–0.80 to –0.28)	–0.02 (–0.37 to 0.34)
Brief pain inventory—Interference	Mean change (95% CI)	Mean change (95% CI)	Mean difference (95% CI)
Week 8	–0.65 (–0.96 to –0.34)	–0.61 (–0.93 to –0.30)	–0.04 (–0.47 to 0.39)
Week 26	–0.58 (–0.90 to –0.26)	–0.78 (–1.10 to –0.45)	0.20 (–0.24 to 0.63)
FACT-Cog PCI subscale	Mean change (95% CI)	Mean change (95% CI)	Mean difference (95% CI)
Week 8	5.44 (3.85 to 7.02)	3.93 (2.31 to 5.54)	1.51 (–0.69 to 3.71)
Week 26	4.66 (3.05 to 6.26)	4.98 (3.33 to 6.63)	–0.32 (–2.57 to 1.92)
PROMIS physical health	Mean change (95% CI)	Mean change (95% CI)	Mean difference (95% CI)
Week 8	2.16 (1.27 to 3.05)	1.84 (0.92 to 2.76)	0.32 (–0.93 to 1.57)
Week 26	1.84 (0.93 to 2.76)	2.41 (1.48 to 3.35)	–0.57 (–1.84 to 0.71)
PROMIS mental health	Mean change (95% CI)	Mean change (95% CI)	Mean difference (95% CI)
Week 8	4.55 (3.60 to 5.49)	3.77 (2.81 to 4.74)	0.77 (–0.53 to 2.07)
Week 26	3.74 (2.77 to 4.70)	4.02 (3.04 to 5.01)	–0.29 (–1.62 to 1.04)

NOTE. For each outcome, estimates are derived from a linear mixed model with baseline means constrained to be equal across study arms. The dependent variable vector included the prerandomization baseline (week 0) assessment and all postrandomization assessments at weeks 4, 8, 16, and 26. The independent variables were the random assignment stratification variables (study site, baseline anxiety medication use, and English/Spanish language preference), treatment arm, week (categorical), and the arm-by-week interaction. A patient-level random intercept was included in the model to account for the repeated outcome measurements within patients. The HADS anxiety subscale contains seven items, each scored on a 4-point Likert scale from 0 to 3; items are summed to calculate the subscale score, which ranges from 0 to 21. Lower scores on HADS, Brief Fatigue Inventory, Insomnia Severity Index, and Brief Pain Inventory indicate improved symptoms. Higher scores on FACT-Cog and PROMIS scales indicate improved symptoms and quality of life.

Abbreviations: CBT, cognitive behavioral therapy; HADS, Hospital Anxiety and Depression Scale; FACT-Cog, Functional Assessment of Cancer Therapy-Cognitive Function; MT, music therapy; PCI, perceived cognitive impairment; PROMIS, Patient-Reported Outcomes Measurement Information System; SD, standard deviation.

^a^ The coprimary end point comparisons of mean differences between the arms in HADS Anxiety scores at weeks 8 and 26 tested whether music therapy was noninferior to CBT. Both these noninferiority comparisons were tested at significance threshold *P* < .025 to limit the overall type I error rate for the primary end point comparisons at *P* < .05. Between-arm comparisons for all other outcome measures were secondary end points and are presented as point estimates with 95% CI. The widths of the 95% CI for secondary outcomes have not been adjusted for multiplicity, and these intervals should not be used to draw definitive conclusions about treatment effects.

^b^ Primary end point comparison of noninferiority of music therapy to CBT at week 8. Point estimate with one-sided 97.5% CI is presented. The HADS Anxiety SD at week 8 was 3.42, and the noninferiority margin (δ) was prespecified as δ = 0.35 × SD = 0.35 × 3.42 = 1.20. This δ lies beyond the one-sided (upper bound) 97.5% CI; therefore, we conclude that music therapy was noninferior to CBT at week 8 at our prespecified *P* < .025 significance threshold. The actual *P* value for this noninferiority test was *P* < .001.

c Primary end point comparison of noninferiority of music therapy to CBT at week 26. Point estimate with one-sided 97.5% CI is presented. The HADS Anxiety SD at week 26 was 3.65, and the noninferiority margin (δ) was prespecified as δ = 0.35 × SD = 0.35 × 3.65 = 1.28. This δ lies beyond the one-sided (upper bound) 97.5% CI; therefore, we conclude that music therapy was noninferior to CBT at week 26 at our prespecified *P* < .025 significance threshold. The actual *P* value for this noninferiority test was *P* < .001.

### Secondary Outcomes

Music therapy and CBT improved fatigue, depressive symptoms, insomnia, pain, cognitive difficulties, and physical- and mental health–related quality of life. Neither intervention demonstrated superiority (Table 2).

### Anxiety Medication Use

Medication diary data were available for 240 (80%) participants. Seventy-nine (32.9%) participants took at least one psychiatric medication at baseline; 32.1% and 30.4% continued taking medications at weeks 8 and 26, respectively. There were no between-group differences at baseline, week 8, or week 26.

### Treatment Adherence and Fidelity

Regarding treatment adherence, 91.2% of the music therapy group and 82.4% of the CBT group completed six of the seven sessions. Of 123 music therapy sessions reviewed for treatment fidelity, 112 (91.1%) were delivered with fidelity. A higher number of CBT sessions were reviewed because of a higher number of CBT interventionists. Of 180 CBT sessions reviewed, 174 (96.7%) were delivered with fidelity.

### Adverse Events

There were three mild adverse events (depressive symptoms, headache) reported in music therapy and one mild adverse event (increased anxiety) in CBT.

### Heterogeneity of Treatment Effects

**FIG A2.**
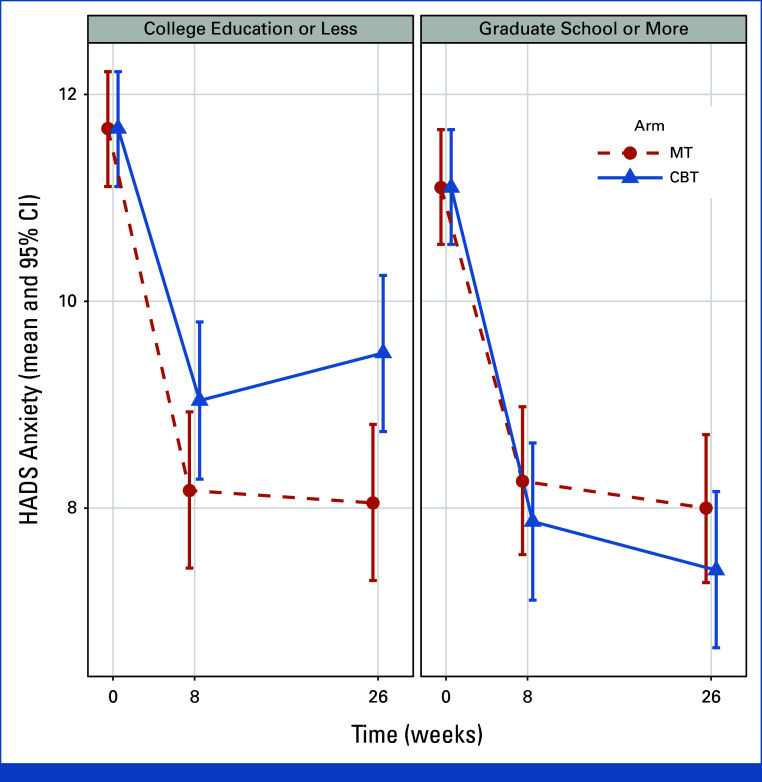
Heterogeneity of the treatment effect by education level. This figure shows the mean HADS anxiety subscale score by treatment group from baseline through week 26 among patients who received college education or less and among patients who received graduate school education or more. CBT, cognitive behavioral therapy; HADS, Hospital Anxiety and Depression Scale; MT, music therapy.

**FIG A3.**
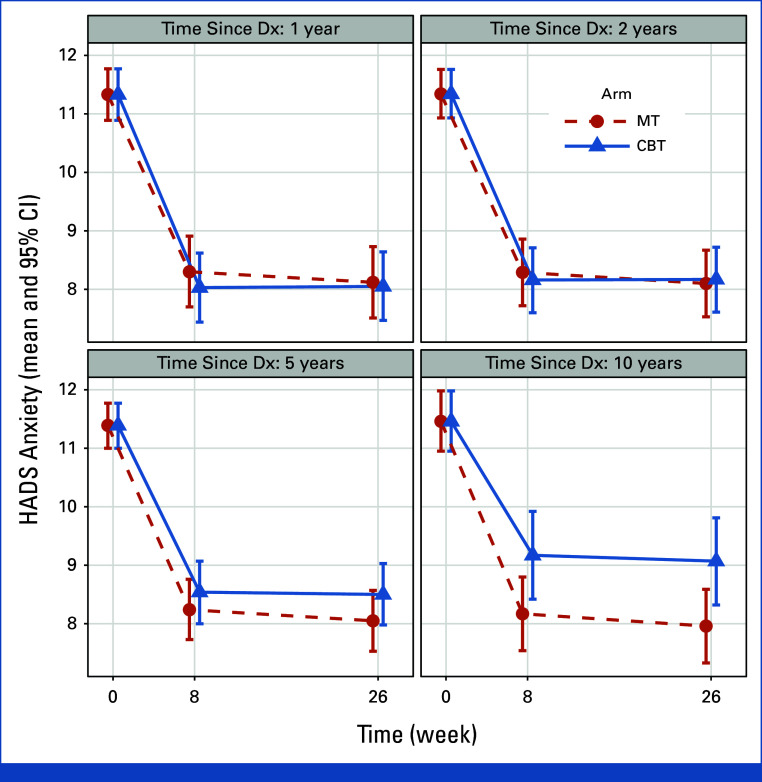
Heterogeneity of the treatment effect by time since cancer diagnosis. This figure shows the mean HADS anxiety subscale score by treatment group from baseline through week 26 among patients who were initially diagnosed with cancer 1, 2, 5, or 10 years ago. CBT, cognitive behavioral therapy; Dx, diagnosis; HADS, Hospital Anxiety and Depression Scale; MT, music therapy.

The effectiveness of music therapy was similar across education levels; however, CBT was more effective among those who completed a graduate or professional degree, compared with those who received only a college degree or less (*P* = .03). The effectiveness of music therapy was similar across years since cancer diagnosis; however, the effectiveness of CBT decreased as the number of years since cancer diagnosis increased (*P* = .03). No heterogeneity of treatment effects was observed with regard to sex, race, ethnicity, or expectancy (Appendix Figs A2 and A3).

## DISCUSSION

Music therapy was noninferior to CBT for anxiety symptoms in survivors of cancer when both were delivered via telehealth. In both groups, approximately 70% of participants reported clinically meaningful improvements, and anxiety reductions persisted up to 26 weeks without maintenance sessions. Both interventions showed similar improvements in fatigue, other comorbid symptoms, and quality of life.

The trial results aligned with meta-analyses demonstrating the effectiveness of CBT, reinforcing its status as a first-line treatment for anxiety.^9^ While the number and frequency of CBT treatments vary in clinical practice, our seven-session weekly intervention is consistent with typical treatment schedules documented in the literature.^53,54^ Given that licensed social workers are the most commonly employed mental health providers in cancer care,^67^ our study accurately reflects the real-world implementation of CBT in oncology. By enrolling Spanish-speaking survivors and using remote delivery, this trial contributes to the growing research on linguistic and telehealth adaptions of CBT, which could help expand treatment access.^39,68^

The trial confirmed the primary hypothesis that music therapy was as effective as CBT for short- and long-term anxiety reduction. In a meta-analysis of music therapy for cancer populations, music therapy was associated with anxiety reduction immediately post-treatment,^27^ but no trials included long-term follow-up or compared music therapy against other first-line anxiety treatments. Our study provides novel findings on the durable effects of music therapy and its comparative effectiveness relative to first-line CBT. The findings of long-term anxiety reduction contrast with a meta-analysis of noncancer patients, which found no sustained benefit.^69^ Most trials in the meta-analysis used receptive activities, such as music listening, designed to provide short-term distractions from negative stimuli.^69^ By contrast, our protocol incorporated active music experiences, specifically collaborative songwriting, which was purported to target social-cognitive factors underlying long-term emotional adjustment.^44^ The growing availability of music therapy at cancer centers and community hospitals,^25,26^ coupled with its capacity for telehealth delivery,^40-42^ underscores its potential scalability as a mental health intervention; however, limited insurance coverage remains a barrier.

In contrast to our secondary hypothesis, music therapy was not superior to CBT with regard to reductions in fatigue. Both interventions for anxiety produced similar improvements in fatigue and other comorbid symptoms, suggesting that anxiety could be a promising therapeutic target for reducing overall symptom burden in survivors of cancer. While our analysis examined each symptom in isolation, anxiety often co-occurs and correlates with other symptoms in survivors of cancer.^3,4^ Therefore, future research should analyze symptoms as clusters to determine whether there are differential treatment effects. This knowledge could help patients and clinicians choose between two interventions that otherwise have similar effects on individual symptoms.

The finding that two interventions with distinct purported mechanisms can produce comparable reduction in anxiety underscores the complex nature of this symptom, with multiple factors contributing to its persistence or resolution.^70^ In addition to modifying thoughts and behaviors via CBT, engaging in creative expression within the supportive social context of music therapy appears to be another effective approach for reducing anxiety in the long term.^44,45^ Future studies should confirm these purported mechanisms and explore which treatment approach is more suitable for different individuals.

In this trial, music therapy was similarly effective for anxiety across years since diagnosis and across education levels, but CBT was less effective as years since cancer diagnosis increased and was more effective among graduate degree holders. Our music therapy intervention provided a creative outlet for processing various types of stressors, whereas our CBT intervention focused on cancer-related distress; this difference could explain why CBT might have been less effective for survivors with life stressors unrelated to cancer as time since diagnosis increased. Given that the HADS anxiety subscale assessed general anxiety symptoms, more research is needed on how these interventions affect cancer-specific anxiety, such as fear of recurrence.^71^ As an experiential intervention involving creative expression, music therapy may be more effective among people of lower educational background than CBT,^72^ a more cerebral intervention involving analytical skills. However, our sample was highly educated, which may preclude a conclusive interpretation. Additional research on how treatment effects differ by patient characteristics may help inform personalized anxiety management.

Our trial has several limitations. First, we enrolled participants with elevated anxiety symptoms rather than anxiety disorders or cancer-specific subtypes of anxiety, such as fear of recurrence. Although sensitivity analyses demonstrated that interventions were similarly effective among survivors with generalized anxiety disorder, the diagnoses of generalized anxiety disorder were based on patient self-report rather than clinician assessment. Second, the study population was mostly female and highly educated, and survivors of breast cancer comprised nearly half of the sample, which affects generalizability. Because of the telehealth format, generalizability may also be limited for those with poor digital access or literacy. Third, CBT had slightly higher attrition and lower adherence than music therapy, which could be due to treatment preferences.^73^ Fourth, we could not account for Hawthorne effects or regression to the mean because of the lack of a usual care control. Given that the primary aim was to determine the comparative effectiveness of two real-world treatments, a placebo or attention control group was not included, precluding evaluation of specific versus nonspecific treatment effects. Finally, we excluded patients who received seven sessions of music therapy or CBT in the preceding 6 months, but we did not track the exact number or type of previous sessions received among eligible participants who enrolled.

Despite these limitations, our trial matched both interventions for therapist time and attention, increasing the rigor of the comparison. An additional strength is enrollment of English- and Spanish-speaking survivors with broad racial and ethnic representation. Other strengths include high treatment adherence, low attrition, long-term follow-up, and real-world applicability. Our standardized treatment protocols were taught to 14 interventionists and delivered with fidelity on a widely available videoconferencing platform, further enhancing reproducibility and scalability. The telehealth format has the potential to expand access to mental health services.

In this randomized clinical trial, music therapy was noninferior to CBT for short- and long-term anxiety reduction among diverse survivors of cancer. Music therapy should be considered alongside first-line CBT to expand treatment options for anxiety during cancer survivorship.

## Data Availability

A data sharing statement provided by the authors is available with this article at DOI https://doi.org/10.1200/JCO-25-00726.

## APPENDIX

**TABLE A1.** Sensitivity Analyses Including Only Patients With Generalized Anxiety Disorder

HADS Anxiety Subscale (primary)	MT	CBT	Between-Group Difference^a^
	Mean Change (95% CI)	Mean Change (95% CI)	Mean Difference (one-sided 97.5% CI)
Week 8	–3.13 (–4.02 to –2.24)	–2.98 (–3.88 to –2.09)	–0.15 (∞ to 1.04)^b^
Week 26	–3.35 (–4.24 to –2.45)	–2.49 (–3.39 to –1.60)	–0.86 (∞ to 0.34)^c^

NOTE. Lower scores on HADS indicate lower anxiety severity. For each outcome, estimates are derived from a linear mixed model with baseline means constrained to be equal across study arms. The dependent variable vector included the prerandomization baseline (week 0) assessment and all postrandomization assessments at weeks 4, 8, 16, and 26. The independent variables were the random assignment stratification variables (study site, baseline anxiety medication use, and English/Spanish language preference), treatment arm, week (categorical), and the arm-by-week interaction. A patient-level random intercept was included in the model to account for the repeated outcome measurements within patients.

Abbreviations: CBT, cognitive behavioral therapy; HADS, Hospital Anxiety and Depression Scale; MT, music therapy; SD, standard deviation.

^a^ The coprimary end point comparisons of mean differences between the arms in HADS Anxiety scores at weeks 8 and 26 tested whether music therapy was noninferior to CBT. Both these noninferiority comparisons were tested at significance threshold *P* < .025 to limit the overall type I error rate for the primary end point comparisons at *P* < .05. Between-arm comparisons for all other outcome measures were secondary end points and were not adjusted for multiple comparisons. Results are presented as point estimates with 95% CI.

^b^ Primary end point comparison of noninferiority of music therapy to CBT at week 8. Point estimate with one-sided 97.5% CI is presented. The HADS Anxiety SD at week 8 was 3.50, and the noninferiority margin (δ) was prespecified as δ = 0.35 × SD = 0.35 × 3.50 = 1.23. This δ lies beyond the one-sided (upper bound) 97.5% CI; therefore, we conclude that music therapy was noninferior to CBT at week 8 at our prespecified *P* < .025 significance threshold. The actual *P* value for this noninferiority test was *P* < .013.

^c^ Primary end point comparison of noninferiority of music therapy to CBT at week 26. Point estimate with one-sided 97.5% CI is presented. The HADS Anxiety SD at week 26 was 3.64, and the noninferiority margin (δ) was prespecified as δ = 0.35 × SD = 0.35 × 3.64 = 1.27. This δ lies beyond the one-sided (upper bound) 97.5% CI; therefore, we conclude that music therapy was noninferior to CBT at week 26 at our prespecified *P* < .025 significance threshold. The actual *P* value for this noninferiority test was *P* < .001.
