# Supplementary material for: Music Therapy Versus Cognitive Behavioral Therapy via Telehealth for Anxiety in Survivors of Cancer: A Randomized Clinical Trial
Source: J Clin Oncol. 2026 Jan 6;44(5):375–85. doi: 10.1200/JCO-25-00726 (PMC12879169; doi:10.1200/JCO-25-00726)
Supplement: Supplementary file 1 [file jco-44-375-s002.pdf]

MSK PROTOCOL COVER SHEET

Music Therapy vs. Cognitive-Behavioral Therapy for Cancer-related Anxiety (MELODY)

Principal Investigator/Department: Jun J. Mao/Medicine

Acronyms

CBT = cognitive behavioral therapy

MT = music therapy

Table of Contents

1.0 PROTOCOL SUMMARY AND/OR SCHEMA 3
2.0 OBJECTIVES AND SCIENTIFIC AIMS 4
3.0 BACKGROUND AND RATIONALE 4
4.0 OVERVIEW OF STUDY DESIGN/INTERVENTION 8
4.1 Design 8
4.2 Intervention 9
5.0 THERAPEUTIC/DIAGNOSTIC AGENTS & NON-THERAPEUTIC ASSESSMENTS 11
6.0 CRITERIA FOR PARTICIPANT ELIGIBILITY 11
6.1 Participant Inclusion Criteria 11
6.2 Participant Exclusion Criteria 12
7.0 RECRUITMENT PLAN 12
7.1 Research Participant Registration 13
7.2 Randomization 14
8.0 INFORMED CONSENT PROCEDURES 14
9.0 PRE-TREATMENT/INTERVENTION 15
10.0 TREATMENT/INTERVENTION PLAN 16
11.0 EVALUATION DURING TREATMENT/INTERVENTION 16
12.0 CRITERIA FOR REMOVAL FROM STUDY 18
13.0 CRITERIA FOR OUTCOME ASSESSMENT AND ENDPOINT EVALUABILITY 19
14.0 BIOSTATISTICS 20
15.0 TOXICITIES/RISKS/SIDE EFFECTS 23
15.1 Serious Adverse Event (SAE) Reporting 24
16.0 PROTECTION OF HUMAN PARTICIPANTS 25
16.1 Privacy 26
16.2 Data Management 26
16.3 Quality Assurance 27
16.4 Data and Safety Monitoring 27
17.0 REFERENCES 28
18.0 APPENDICES 37

1.0 PROTOCOL SUMMARY AND/OR SCHEMA

Cancer survivors represent one of the fastest growing populations in the United States (U.S.).[1] Unfortunately, nearly one in three survivors suffer from anxiety symptoms as a long-term consequence of cancer and its treatment.[2-7] Characterized by restlessness, muscle tension, and worry, anxiety symptoms worsen quality of life[8-11] and impair daily functioning through associations with poor sleep,[12] depressed mood,[13-15] fatigue,[16-19] pain,[20] and cognitive dysfunction.[21] Although pharmacological treatment options are available, polypharmacy has become a growing concern for cancer survivors.[22-25]

Music therapy (MT)[26-29] and cognitive behavioral therapy (CBT)[30-32] are non-pharmacological treatments that have demonstrated effectiveness for anxiety symptoms in cancer populations. CBT is widely considered a first-line treatment for anxiety,[33-35] and the American Society of Clinical Oncology (ASCO)[36,37] and the National Comprehensive Cancer Network (NCCN)[38] recognize CBT as first-line anxiety treatment in patients with cancer. However, not all individuals respond or wish to pursue CBT.[39-42] MT is associated with moderate effect sizes for anxiety relative to usual care[26] and is recommended by ASCO,[36,37] NCCN,[38] and the Society for Integrative Oncology (SIO)[43,44] as a treatment option for anxiety in cancer populations. Further, research suggests that participation in active MT techniques (e.g. singing, playing instruments, creating songs), is associated with greater reductions in fatigue, compared with exposure to receptive MT approaches (e.g. music-guided relaxation).[45] Despite strong support for both CBT and MT, it remains unclear how MT compares to CBT as a treatment alternative for anxiety and associated symptoms (e.g. fatigue).

The need for effective, accessible mental health treatment has become even more critical in the midst of COVID-19.[46] The pandemic has also accelerated the digital transformation of healthcare and society at large, forcing patients and clinicians to navigate a growing array of remotely delivered care options.[47] Based on our preliminary studies, MT and CBT have demonstrated the capacity for virtual delivery and scalability, but their comparative effectiveness in a virtually delivered format remains unknown. Further, cancer survivors' experiences of CBT and MT have not been examined, a notable gap given that not all survivors prefer or respond to the same treatments.

To guide treatment decision-making in the new digital healthcare era, we propose the **M**usic Therapy vs. Cognitive B**e**haviora**l** Therapy f**o**r Cancer-relate**d** Anxiet**y** (**MELODY**) trial. The MELODY trial is a two-arm, parallel group, randomized clinical trial (RCT) to compare the effectiveness of virtually delivered MT versus CBT for anxiety (primary outcome) and co-morbid symptoms of depression, fatigue, insomnia, pain, cognitive dysfunction, and quality of life (secondary outcomes) in a diverse, heterogeneous sample of 300 English or Spanish-speaking cancer survivors (Figure 1). Participants will be recruited at MSK and participating institutions. Participants will be randomized to receive seven weekly 60-minute treatments of either MT or CBT delivered virtually over seven weeks. Participants will complete study assessments prior to randomization (week 0) and at weeks 4, 8, 16, and 26. A subgroup of

60 survivors will complete optional semi-structured interviews after treatment to understand their unique experiences with MT or CBT. All participants will be followed for 26 weeks.

Figure 1.
Study Schema.

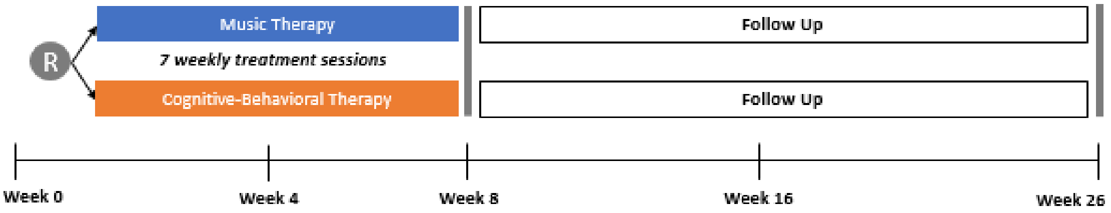

2.0 OBJECTIVES AND SCIENTIFIC AIMS

- Aim 1: To determine the comparative effectiveness of virtual MT versus virtual CBT for anxiety and co-morbid symptoms in cancer survivors.
  - Hypothesis 1(a): MT will be non-inferior to CBT for treating anxiety symptoms among survivors at week 8 (end of treatment) and week 26 (long-term follow-up).
  - Hypothesis 1(b): Compared with CBT, MT will be associated with significantly greater improvement in fatigue co-occurring with anxiety.
  - Exploratory Hypothesis: Survivors may have unique experiences with MT and CBT for anxiety during cancer survivorship.
- Aim 2: To identify patient-level factors associated with greater anxiety symptom reduction for MT and CBT.
  - Hypothesis 2: Specific socio-demographic characteristics (e.g. age, sex, race, education) or psychological attributes (i.e. expectancy) will be associated with treatment response to MT or CBT.

3.0 BACKGROUND AND RATIONALE

3.1. The Burden of Anxiety During Cancer Survivorship. With advances in oncology care, the number of cancer survivors in the U.S. is expected to increase dramatically and exceed 22 million by the end of this decade.[1] This accelerating trend highlights the public health importance of addressing the long-term consequences of cancer in the rapidly growing survivor population. In its report entitled "Cancer Care for the Whole Patient," the National Academy of Medicine recognized psychosocial services as an essential component of high-quality cancer care.[48] A *Lancet Oncology* meta-analysis identified anxiety as one of the most common mental health issues facing cancer survivors.[49] Compared with the general population, cancer survivors experience higher rates of anxiety,[3,7,49,50] and up to a third suffer from clinically significant anxiety symptoms.[2-7] Characterized by restlessness, muscle tension, and worry that is difficult to control, anxiety is a highly disruptive symptom,

associated with poor sleep,[12] depressed mood,[13-15] and fatigue.[16-19] Anxiety also contributes to poor treatment adherence[51-53] and increased healthcare expenditures.[54,55] In fact, anxiety is the primary driver of poor quality of life among cancer survivors[8-11] and therefore represents an important treatment priority during cancer survivorship.

Although pharmacological treatment options for anxiety are available,[36] polypharmacy is a growing concern among cancer survivors.[22-25] Compared with the general population, cancer survivors have much higher rates of polypharmacy, with 64% taking five or more unique medications.[24] A third of survivors receive at least one psychotropic medication (e.g. anxiolytic, antidepressants), and nearly one in six report using two or more classes of psychotropic medications.[25] Psychotropic polypharmacy is associated with poor quality of life, financial toxicity, and higher risk of side effects and drug interactions.[25] According to ASCO clinical guidelines, “Caution is warranted with respect to the use of benzodiazepines in the treatment of anxiety, specifically over the longer term. These medications carry an increased risk of abuse and dependence and are associated with adverse effects.”[36] These challenges of polypharmacy illustrate the need for non-pharmacological treatment options for anxiety during cancer survivorship.

3.2. Cognitive Behavioral Therapy: A Highly Effective Anxiety Treatment for Some, but Not All, Individuals. Cognitive behavioral therapy (CBT) is an evidence-based, non-pharmacological intervention delivered by licensed mental health providers.[33,35] Informed by the cognitive behavior model of anxiety, CBT focuses on the relationship between thoughts, behaviors, and emotions and how thoughts and behaviors can exacerbate or reduce anxiety.[56-58] The therapeutic components of CBT consist of psycho-education on anxiety, relaxation techniques, cognitive restructuring, and strategies for planning activity engagement and managing realistic worries.[59,60] These components target the somatic symptoms of anxiety (e.g. muscle tension), as well as the thoughts (e.g. “What if my next scan is bad?”) and behaviors (e.g. excessive symptom monitoring) that trigger and exacerbate anxiety. CBT is widely recognized as an effective, first-line therapy for anxiety in the general population.[33-35] ASCO and NCCN recognize CBT as first-line anxiety treatment in patients with cancer.[36,38] Meta-analyses demonstrate moderate-to-large effects of CBT on anxiety symptoms in cancer patients and survivors relative to control conditions, with effect sizes ranging from 0.42-1.10.[30,32,61,62]

Despite the substantial evidence base of CBT, studies have demonstrated that 20-25% of CBT participants fail to complete a full treatment course (defined as completion of all intervention sessions or modules)[40,63] and 37% do not achieve clinically meaningful improvement in anxiety symptoms.[39,64] To inform the design of our current study, we conducted an engagement session with seven survivors of various cancer types. During the engagement session, those with prior CBT experience commented that a full treatment course requires “significant mental and emotional stamina” that may be too demanding and taxing for some survivors. Some patient stakeholders also noted the social stigma surrounding psychotherapy in certain cultures. Research showed that underrepresented racial/ethnic groups are more likely than whites to delay, avoid, or drop out of mental health

treatments.[41,42] These challenges of CBT highlight the importance of studying other non-pharmacological treatments that are not only effective but also culturally acceptable to diverse cancer survivors.

3.3. Music Therapy: An Evidence-based Anxiety Treatment with Increasing Availability at Cancer Centers. MT is a non-pharmacological, evidence-based intervention, in which board-certified music therapists engage patients in personally tailored experiences with music to achieve therapeutic goals.[65] These experiences range from music-guided relaxation to more active forms of musical engagement, including singing and improvising music.[65] The social-cognitive processing model of emotional adjustment of cancer informs the use of MT for anxiety management in cancer survivors.[66] Supported by empirical studies,[67,68] this model conceptualizes the cancer treatment journey as a disruptive, trauma-like experience that must be cognitively processed in a supportive social context for healthy emotional adjustment to take place.[66,67] Growing research demonstrates the capacity for MT to influence these social-cognitive processes central to anxiety. Interactive music experiences have been shown to build social connections and promote a sense of belonging.[69-71] Prior studies also suggest that MT provides novel, creative outlets (e.g. through songwriting) to cognitively process past traumatic experiences that may otherwise be difficult to verbalize.[72,73] Indeed, these findings were echoed by our patient stakeholders, many of whom used music to cope with difficult emotions during their cancer journeys. In addition to its social-cognitive effects, musical engagement has been shown to modulate brain regions (i.e. amygdala)[69] and biological systems (i.e. hypothalamic-pituitary-adrenal axis,[74-76] autonomic nervous system[77-80]) responsible for emotional regulation and implicated in anxiety and mood disorders.[81-84] Importantly, ethnographic and phylogenetic research has identified music as a defining characteristic of humankind across all cultures around the world.[85,86] The universality of music supports the potential of MT to resonate with a diverse cancer survivor population.

MT has a growing evidence base for cancer symptom management[26-29] and is recommended by ASCO,[37] NCCN,[38] and SIO[43,44] as a treatment option for anxiety in cancer populations. In a recent meta-analysis of 20 studies (N=1,565), MT was associated with a moderate reduction in anxiety among cancer patients, compared with usual care (Cohen's *D*=0.65, 95% Confidence Interval [CI] 0.11-1.20).[27] These findings are consistent with an earlier Cochrane review conducted by our study co-investigator Dr. Bradt (52 trials, N=3,731) that found a similar magnitude of anxiety reduction from MT compared with usual care (*D*=0.71, 95% CI 0.43-0.98).[26] From 2009 to 2016, the number of cancer centers providing information about MT on public-facing websites increased significantly from 21 (51.2%) to 37 (82.2%).[87] By 2016, 22 (48.9%) of the then 45 National Cancer Institute (NCI)-designated Comprehensive Cancer Centers offered MT services.[87] In our recently updated review of NCI-designated Comprehensive Cancer Centers and 100 community-based cancer programs, we found the percentage of NCI-designated Comprehensive Cancer Centers offering MT increased considerably to 74.1%; MT services were also available at 55% of community-based cancer programs.[88] Due to its growing evidence base and availability, MT represents a promising alternative to CBT for the treatment of anxiety.

3.4. Digital Transformation of Healthcare in the Midst of COVID-19: A Timely Opportunity to Improve Access to Music and Cognitive Behavioral Therapies. In the past two decades, the percentage of U.S. adults who use the internet has increased from 52% to 90%.[89] Approximately 59-79% have access to home broadband internet services,[89] and 37% use smartphones as their primary access to the internet.[90] Cancer centers across the country are increasingly leveraging these digital trends to monitor and manage symptoms remotely.[91] The COVID-19 pandemic has accelerated the adoption of digital platforms for care delivery, and use of videoconferencing for telehealth services has increased by 8,700%.[47] Videoconferencing use has also increased dramatically outside of the healthcare setting, as people rely on this technology to work remotely and stay socially connected. As an exemplar of this trend, Zoom has become one of the most widely used videoconferencing platforms worldwide with over 300 million daily meeting participants.[92]

Although key disparities in digital access and literacy remain,[93] the widespread adoption of videoconferencing offers a promising platform to reduce barriers to mental health services. Therapists have successfully used videoconferencing to deliver MT remotely to military populations,[94-98] in hospital settings,[99,100] and to patients with autism.[101] At the outset of the pandemic, the American Music Therapy Association (AMTA) and other MT professionals rapidly developed and implemented telehealth guidelines and resources to facilitate the virtual delivery of MT services to patients isolated at home.[99,102] There is an extensive body of research (>100 trials) on internet-delivered CBT interventions,[103] and studies have consistently demonstrated that CBT delivered remotely is as effective and acceptable as in-person treatments for anxiety.[104] Thus, both MT and CBT are equipped for this new, evolving digital healthcare landscape, with unique potential for scalability to reduce barriers to mental health services.

3.5. Gaps in the Evidence: Decisional Dilemmas Facing Patients and Providers in the New Digital Healthcare Landscape. Although CBT is widely considered a first-line treatment for anxiety, not all individuals are able to complete a full treatment course[40,63] or achieve meaningful improvements in their symptoms.[39,64] People may also be reluctant to pursue CBT due to the socio-cultural stigma surrounding psychotherapy in different communities.[41,42] For individuals who do not respond or wish to pursue CBT, it remains unclear whether MT is an effective treatment option that is non-inferior to CBT. To inform treatment decision-making, the American Psychological Association (APA), in its Resolution on the Recognition of Psychotherapy Effectiveness, has called for “continued and further research on the comparative effectiveness” of CBT and other psychotherapeutic interventions.[105] Another key evidence gap is the lack of diverse representation in CBT trials. The majority of CBT trial participants to date have been well-educated and white, leading to significant uncertainty on whether CBT can adequately address the mental health needs of under-represented populations. According to the APA, “current psychotherapy research suggests that racial/ethnic minorities, those with low socioeconomic status, and members of the LGBT [lesbian, gay, bisexual, and transgender] community may face specific challenges not addressed by current evidence-based treatment.”[105] In a similar vein, the NCCN guidelines highlight that “psychosocial interventions have disproportionately targeted women

with breast cancer… [and] more interventions targeting patients with other cancer types, or inclusion of mixed cancer types, should be developed and evaluated."[38] Inclusion of underrepresented groups in clinical trials is necessary to advance mental health treatment for a diverse cancer survivor population. Finally, CBT research has compared remote delivery versus traditional in-person formats, but a paucity of studies has compared virtually delivered CBT with other virtually delivered interventions, such as MT.[103,104] As healthcare continues to undergo rapid digital transformation, patients and providers will be faced with a vast and growing array of remote care options. In their systematic review of digital health interventions for cancer survivors, Harris et al. emphasized that "one size may not fit all" and there is a "need to identify who is most likely to benefit from digital interventions."[91] Comparative effectiveness research on different virtually delivered treatments (e.g. CBT versus MT) is essential to guide timely and patient-centered decision-making and help patients and providers navigate this new digital healthcare landscape.

3.6. Summary of Significance. Anxiety is a highly prevalent and distressing symptom in the rapidly growing cancer survivor population.[49] Both MT[26-29] and CBT[30-32] are effective for anxiety symptoms and recommended by leading professional cancer societies, including ASCO,[36,37] NCCN,[38] and SIO;[43,44] however, their comparative effectiveness remains unknown. The MELODY study is the first and largest clinical trial to compare the short- and long-term effectiveness of MT versus CBT for anxiety in cancer survivors. As the world grapples with the devastating psychological consequences of the COVID-19 pandemic,[46] our findings will help patients, caregivers, and providers make informed decisions regarding these two evidence-based treatments for anxiety. The pandemic has also shone a light on the preexisting mental health disparities affecting underrepresented racial/ethnic communities.[41,42] The MELODY study will enroll a racially/ethnically diverse, heterogenous population from urban, suburban, and rural settings to ensure that findings are applicable to diverse cancer survivors. Finally, by focusing on virtually delivered MT and CBT, the MELODY study will capitalize on the accelerating digital trends in healthcare and society at large, resulting in timely findings on two evidence-based, scalable, and accessible interventions with unique potential to address the unmet mental health needs of cancer survivors.

4.0 OVERVIEW OF STUDY DESIGN/INTERVENTION

4.1 Design

The proposed MELODY trial is a two-arm, parallel group, randomized clinical trial (RCT) to compare the effectiveness of MT versus CBT for anxiety and co-morbid symptoms in a diverse, heterogeneous sample of 300 cancer survivors (Figure 1). We chose an RCT design because it is the most appropriate way to obtain a valid measure of effectiveness, controlling for the large number of variables, whether known or unknown, measurable or immeasurable, that may confound the association between exposure and the primary outcome. The study is guided by the PICOTS (Population, Interventions, Comparator, Outcomes, Time, Setting)

framework, which is designed to inform development of a research question and rigorous study methods.[106]

- Population: We focused on cancer survivors because they represent one of the fastest growing groups with a significant burden of anxiety and co-morbid symptoms.
- Intervention/Comparator: We chose to evaluate MT and CBT, based on their demonstrated effectiveness for anxiety symptoms, their capacity for virtual delivery, their endorsement in clinical guidelines of professional cancer societies, and their wide acceptance among cancer survivors. Given that CBT is widely recognized as a first-line therapy for anxiety, we decided to test the non-inferiority of MT to CBT, with the goal of understanding whether MT can be an appropriate treatment option for patients who lack CBT access or do not wish to pursue CBT. To provide complementary information for treatment decision-making, we will also test which intervention is superior for addressing fatigue and other symptoms co-occurring with anxiety.
- Outcome: We chose to study anxiety symptoms, rather than an anxiety disorder. Research indicates anxiety symptoms are associated with poor quality of life and other problematic outcomes in cancer populations, even if symptoms do not meet criteria for an anxiety disorder.[8,107] Further, cancer care guidelines (e.g. American College of Surgeons Commission on Cancer) are often based on symptom severity, rather than psychiatric diagnoses.[108] Thus, by focusing on symptoms, our study will align with clinical guidelines and capture more patients who could potentially benefit from interventions.
- Time: We planned for long-term follow-up of 26 weeks because interventions such as MT and CBT involve long-term practice of self-management skills. There is also significant interest among cancer survivors in durability of treatment effects.
- Setting: Our decision to focus on virtual delivery of MT and CBT was informed by the accelerating digital trends within healthcare, as well as by the critical need to reduce barriers to mental health services during and beyond the COVID-19 pandemic.

4.2 Intervention

We selected MT and CBT as the interventions/comparators based on their demonstrated effectiveness for anxiety symptoms, their capacity for virtual delivery, their endorsement in clinical guidelines of professional cancer societies (ASCO, NCCN, and SIO), and their wide acceptance among cancer survivors. Although the duration of treatments may vary in private practice settings, we have designed the MT and CBT interventions to have equal patient-therapist contact time to ensure rigorous comparative effectiveness comparison. Patients will receive seven weekly 60-minute treatments over seven weeks. All sessions will be delivered virtually through Zoom's HIPAA-compliant, encrypted, passcode-protected videoconferencing software. If the Zoom platform has logistical issues (e.g., the audio is not working), we will attempt to troubleshoot the issues over the telephone. If the issues cannot be resolved, the session will be conducted over the telephone. Immediately following session completion, the study team will contact the patient to solve the issue prior to the next session.

4.2.a. Music Therapy. The MT protocol (Appendix A) is based on the neuroscience of music,[69,109] the social-cognitive processing model,[66,67] the scientific literature on MT,[26] and our own clinical and research experience.[110] Healthy psychological adjustment during cancer survivorship requires the processing of feelings and thoughts related to one's cancer journey in a supportive context.[66,67] Therefore, we have structured the intervention protocol to gradually build patient-therapist rapport and allow for greater emotional expressivity and deeper cognitive processing. Guided by the social-cognitive processing model,[66,67] the first few sessions will focus on building a trusting relationship with the therapist, whereas later sessions will focus on cognitive processing of cancer-related experiences and current fears, worries and hopes, using playlist creation and songwriting as an outlet for reflection, expression, and meaning-making. Research also suggests that patients prefer receptive modes of engagement during initial exposure to music therapy.[29,111] As such, the early sessions will focus on receptive techniques (e.g. music-guided stress management) and later sessions will progress to more active music therapy techniques (e.g. songwriting). The protocol includes homework activities in between sessions to promote self-management skills (e.g. use of music-guided deep breathing), strengthen social connections (e.g. sharing of meaningful song with loved ones), provide outlets for cognitive processing (e.g. composing song lyrics), and serve as transitions to the subsequent sessions. Patients will receive a workbook with materials for each session (Appendix H). The MT interventionists will be board-certified music therapists.

4.2.b. Cognitive Behavioral Therapy. The CBT protocol (Appendix B) is based on the scientific literature,[33,35] as well as our clinical and research experience.[112] CBT therapists will include psychologists, social workers, and trainees in clinical psychology doctoral programs and Masters-level social work programs, as reflective of real-world practice and implementation of CBT in oncology settings.[113] In addition to psychologists, we included social workers as CBT interventionists because they are the most commonly employed mental health providers in cancer care.[113] To expand the pool of eligible therapists and enhance future intervention dissemination, the social workers administering CBT will not be required to have prior CBT training or experience in oncology care. Informed by the cognitive behavior model of anxiety, CBT focuses on the relationship between thoughts, behaviors, and emotions and how thoughts and behaviors can exacerbate or reduce anxiety.[56-58] The protocol consists of psycho-education on anxiety, relaxation techniques, and cognitive restructuring, and strategies for planning activity engagement and managing realistic worries.[59,60] These components target the somatic symptoms of anxiety (e.g. muscle tension), as well as the thoughts (e.g. "What if my next scan is bad?") and behaviors (e.g. excessive symptom monitoring) that trigger and exacerbate anxiety. Once identified, these problematic thoughts and behaviors are replaced with thoughts and behaviors that prevent and reduce anxiety. Each session will follow a consistent format that includes an overview of session content, review of the homework exercise from the prior session, information on a new skill for managing anxiety, discussion of the upcoming homework exercise, and a plan for completing the exercise before the next session. Patients will receive a workbook with materials for each session (Appendix I).

4.2.c. Interventionist Training and Treatment Fidelity. All interventionists will be trained about the specific research protocol and educated on the importance of adherence to protocol methods. Study interventionists will be either English-speaking or bilingual in English and Spanish. We will ensure adequate number of bilingual interventionists are available for Spanish-speaking patients. Study interventionists will receive training and supervision from the Lead Therapists remotely to reduce travel burden and simulate remote delivery of the interventions. Training will include didactic information on anxiety in cancer survivors and review of intervention materials. Interventionists will be provided with ongoing supervision over the course of the trial. During the trial, CBT and MT sessions will be recorded and stored on secure, encrypted MSK servers. To ensure that study therapists adhere to the treatment protocol, document treatments appropriately, and maintain fidelity to the core functions of the intervention, the Lead Therapists will review approximately two recordings for each study therapist per week, but the frequency of monitoring may change, depending on the circumstances. For example, the frequency may increase when a new therapist joins the study, and it may decrease if a therapist has already demonstrated adherence to the protocol on a consistent basis. During this review, the Lead Therapists will use treatment fidelity checklists outlining the core intervention components. Deviations from the MT or CBT protocols will be discussed with study therapists during supervision meetings, and strategies will be suggested for minimizing the number of deviations. Therapists who fail to adhere to at least 80% of the treatment fidelity checklist items will be retrained. If a new therapist joins the study protocol, their respective Lead Therapist will train them. In our MT[110] and CBT trials[112,114] of cancer patients, we used similar treatment fidelity strategies and successfully delivered complex interventions with minimal protocol deviations.

Session recording will be optional for Miami Cancer Institute participants.

5.0 THERAPEUTIC/DIAGNOSTIC AGENTS & NON-THERAPEUTIC ASSESSMENTS

MT and CBT are non-pharmacological interventions that do not involve the use of any therapeutic agents or devices.

6.0 CRITERIA FOR PARTICIPANT ELIGIBILITY

We established broad eligibility criteria to be consistent with a pragmatic design while ensuring participant safety and scientific rigor.

6.1 Participant Inclusion Criteria

- English- or Spanish-speaking
- 18 years or older
- Prior cancer diagnosis of any type or stage
- Free of oncological disease, or stable disease with no evidence of progression

- Score of ≥8 on the anxiety subscale of the Hospital Anxiety and Depression Scale (HADS)
- Report anxiety symptoms lasting at least one month
- Willing to adhere to all study-related procedures, including randomization to one of two treatment arms: MT or CBT
- Access to Zoom and a quiet/private location

6.2 Participant Exclusion Criteria

- Completed active cancer treatment (e.g., surgery, radiation, chemotherapy) less than one month prior to enrollment (maintenance hormonal or targeted therapies are allowed).
- Active suicidal ideation, bipolar disorder, schizophrenia, or substance abuse
- Score of ≥10 indicative of cognitive impairment on the Blessed Orientation-Memory-Concentration
- Received a treatment course of seven or greater MT or CBT sessions for anxiety symptoms within the last six months
- Unable to provide informed consent for themselves

7.0 RECRUITMENT PLAN

We plan to recruit the majority of patients at OneMSK locations. Given that this virtually conducted study will include both English- and Spanish-speaking survivors, we will recruit a target sample of N=50 participants from the Miami Cancer Institute (MCI) in South Florida (MSK Cancer Alliance member) to enhance Hispanic accrual. We aim to recruit a total sample of N=20 participants from Drexel University. We aim to enroll a diverse sample that includes traditionally under-represented sociodemographic groups (target accruals: n=60 Hispanic or Latino; n=60 Black; n=20 Asian; n=24 more than one race). This diverse representation will enable us to identify and perform careful exploration of differences between participant subgroups (e.g. race, ethnicity) to inform personalized decision-making.

We will use MSK's Dataline queries to identify survivors who meet basic eligibility criteria. We will then mail a recruitment letter to these potentially eligible patients. The recruitment letter will introduce the study and include instructions for interested patients to contact the clinical research coordinator (CRC). The letter will also provide patients with an opt-out phone number and study e-mail address to contact if they do not wish to participate or be contacted further.

In addition to sending recruitment letters, potential participants can be identified and referred to the study research staff for accrual and consent by study co-investigators. For example, at the MCI site, Dr. Currier will oversee the identification and recruitment of potentially eligible survivors. Dr. Currier is the Director of the Cancer Patient Support Center, where psycho-oncology and integrative medicine services are unified under her leadership. Survivors are routinely referred to her service for anxiety management, so she and her staff will be well-

positioned to identify potentially eligible patients.. MSK CBT therapists will conduct treatment sessions for patients consented at Drexel University. Music therapy sessions for Drexel University patients will be conducted by MSK and/or Drexel University staff. Music therapy and CBT sessions for MCI patients will be conducted by MSK and/or MCI staff. In addition, the PI and other members of the research team will reach out to their MSK colleagues to introduce the study and schedule presentations about the study at Service meetings. Colleagues in Psychiatry/Behavioral Sciences and Survivorship will also be informed about the study, and recruitment materials will be provided to them. Patient screening may be directed by the MSK Counseling Center triage staff. During routine calls to the patients, the Counseling Center staff will obtain approval from the patient to be called for study participation. When approval is confirmed, the Counseling Center staff will send a MSKSecure email to the study CRC with relevant patient information. Study staff may call each approved patient to discuss the study and perform informed consent if the patient is amenable to participating. In addition to Integrative Medicine physicians, other Integrative Medicine therapists can refer patients to the study. Potential participants may also be self-referred or referred by a clinician from other hospitals. Information about the protocol will appear in lay language on MSK's web site and on clinicaltrials.gov. Permission from the clinic sites will be obtained before posting in any location. Materials will also be distributed to referral sources who we have worked with on other research studies. These materials include a recruitment brochure (Appendix J, JJ, JJJ, JJJJ, and JJJJJ), which may be given to potential participants to provide them with a brief summary of the study and study contact information. The different versions of the recruitment brochures will be distributed based on the demographic groups we are targeting to ensure diverse representation in our study population. These brochures will be emailed to the listservs of cancer advocacy groups Harlem Share, Red Door Community, SHARE Cancer Support, Male Care, and Prostate Health Education Network. All study recruitment materials will be submitted to, and approved, by the Institutional Review Board.

Initial contact with potential participants will typically be made by a member of the study team. The recruitment process presents no more than minimal risk to patient privacy, and minimal PHI will be maintained on screening logs. For these reasons, we seek a (partial) limited waiver of authorization to: (1) review MSK patient medical records to identify potential research subjects and obtain information relevant to the enrollment process; (2) converse with patients regarding possible enrollment; (3) handle PHI contained in those records and provided by potential subjects; and (4) maintain minimal PHI information in a screening log of patients approached.

Study staff will schedule interested and potentially eligible patients for an Initial Screening visit and then a Baseline Visit with a study clinician via Zoom's HIPAA-compliant platform to confirm eligibility. If deemed eligible, the clinicians will explain the study procedures and perform informed consent. Once consented, participants will complete the baseline assessment within two weeks from enrollment.

7.1 Research Participant Registration

We will confirm eligibility as defined in the section entitled Inclusion/Exclusion Criteria. We will obtain informed consent by following procedures defined in section entitled Informed Consent Procedures. During the registration process, registering individuals will be required to complete a protocol-specific Eligibility Checklist. The individual signing the Eligibility Checklist is confirming whether the participant is eligible to enroll in the study. Study staff are responsible for ensuring that all institutional requirements necessary to enroll a participant to the study have been completed. See related Clinical Research Policy and Procedure #401 (Protocol Participant Registration).

7.2 Randomization

Patients will be randomized using MSK's Clinical Research Database (CRDB), a secure computer system that ensures full allocation concealment. After eligibility is established and consent is obtained, patients will be registered through the Clinical Trials Management System (CTMS) and then randomized using the Randomization Module in the CRDB. Randomization will be 1:1 (MT: CBT) using randomly permuted blocks of random length, stratified by current anxiety medication use (yes/no), preferred language (English/Spanish), and study site (MSK/MCI). The PI and biostatisticians will be blinded to treatment allocation.

8.0 INFORMED CONSENT PROCEDURES

The consent form/research authorization meets the requirements of the Code of Federal Regulations and the Institutional Review Board/Privacy Board of this Center. The consent form will include the following:

1. The nature, objectives, potential risks, and benefits of the intended study.
2. The length of study, what it entails, and the likely follow-up required.
3. Alternatives to the proposed study.
4. The name of the investigator(s) responsible for the protocol.
5. The right of the participant to accept or refuse study interventions/interactions and to withdraw from participation at any time.
6. How the participants' data will be protected, who will have access to their PHI, and what data will be disclosed for research purposes.

Prior to inclusion in the study and before protocol-specified procedures are carried out, consenting professionals will explain the details of the protocol to participants. Participants will also be informed that they are free to withdraw from the study at any time. The consent discussion may occur in person or remotely via teleconference, telephone, or videoconference.

If a verbal consent is being conducted, the consenting professional will use the IRB/PB approved verbal informed consent script when calling patients. A verbal consent would be used in cases where individuals do not have access to a computer and those who are unable to consent in person, or remotely via teleconference or videoconferenceThe consenting

professional must sign an IRB/PB-approved consent /research authorization script to document the consent discussion and the participant’s agreement.

In following the Code of Federal Regulations Title 45, Part 46, Subpart A, the IRB is waiving the requirement for an investigator to obtain a signed consent form from the participant as the research:

- presents no more than minimal risk of harm to participants, and
- involves no procedures for which written consent is normally required outside of the research context.

In following the Code of Federal Regulations Title 45, Part 164, Subpart E, IRB/PB is waiving the requirement for the investigator to obtain signed research authorization from the participant as:

- the use or disclosure of the PHI involves no more than minimal risk to the privacy of the individuals, based on the following elements:
    - An adequate plan to protect identifiers from improper use and disclosure;
    - An adequate plan to destroy the identifiers at the earliest opportunity consistent with conduct of the research (unless there is a health or research justification for retaining the identifiers, or such retention is otherwise required by law); and
    - Adequate written assurances that the PHI will not be reused or re-disclosed to any other person or entity, except as required by law, for authorized oversight of the research project, or for other research for which the use of disclosure of PHI would be permitted by HIPAA.
- The research could not be practicably conducted without access to and use of the PHI.
- The research could not practicably be conducted without the waiver.

9.0 PRE-TREATMENT/INTERVENTION

9.1. Initial Screening

All potential participants will undergo an initial screening with a CRC in person or over the telephone or Zoom. At this initial contact, research staff will explain the study goals and procedures and the CRC will ensure that participants meet basic eligibility criteria. If participants are potentially eligible, they will be scheduled for a baseline eligibility visit.

9.2. Baseline Visit

Interested and potentially eligible patients will be seen by a clinician (physician, nurse/nurse practitioner, physician assistant, or Integrative Medicine clincians) during a baseline visit to confirm eligibility. This visit can be virtual. If deemed eligible, study staff will explain the study and review the written informed consent with the patient. After informed consent is obtained,

patients will complete baseline assessments within two weeks from enrollment. After baseline assessments are completed, patients will undergo randomization. Please refer to Table 1 for additional details on the assessments collected at the baseline visit.

10.0 TREATMENT/INTERVENTION PLAN

Patients will receive seven weekly 60-minute treatments of MT or CBT over seven weeks (+ four week window). All sessions will be delivered virtually through Zoom's HIPAA-compliant, 256-bit TLS encrypted, passcode-protected videoconferencing software. See Appendices A and B for details on the seven-week intervention plan.

11.0 EVALUATION DURING TREATMENT/INTERVENTION

11.1. Primary Outcome

We will assess anxiety symptoms (primary outcome) using the seven-item anxiety sub-scale of the Hospital Anxiety and Depression Scale (HADS). The reliability, validity, and factor structure of the HADS anxiety sub-scale has been established in cancer patients with a Cronbach's α of 0.83.[115,116] A score of ≥8 indicates presence of anxiety symptoms. Research has identified a minimal clinically important difference (MCID) of 1.7 points.[117]

11.2. Other Patient-Reported Outcomes

Given that anxiety is strongly associated with depressed mood,[13-15] fatigue,[16-19] insomnia,[12] pain,[20] and cognitive dysfunction,[21] we will assess these co-morbid symptoms using validated instruments. We will use the HADS depression subscale (Cronbach's α 0.79) to assess depressive symptoms.[115,116] We will assess fatigue using the Brief Fatigue Inventory (BFI), a nine-item, reliable measure of fatigue, validated in cancer populations with Cronbach's α of 0.96.[118] We will assess insomnia symptoms with the Insomnia Severity Index (ISI),[119] a seven-item instrument validated in cancer populations with Cronbach's α of 0.90.[120] We will use the Brief Pain Inventory (BPI) to assess pain severity and pain-related interference. The BPI has been demonstrated to be a reliable, valid, and responsive measure with a Cronbach's α pf 0.77 to 0.91.[121] We will assess cognitive difficulties with the Functional Assessment of Cancer Therapy – Cognitive Function (FACT-Cog), version 3, a 37-item questionnaire with 4 subscales: perceived cognitive impairments, impact on quality of life, comments from others, and perceived cognitive abilities. The FACT-Cog is a reliable instrument validated in cancer populations with a Cronbach's α of 0.89.[122] Of note, the FACT-Cog instrument will only be administered to participants who reply "YES" to the following question at Week 0: "Are you experiencing difficulties with memory, concentration, or other cognitive abilities?"

Given that anxiety is also a key determinant of quality of life in cancer survivors,[8-11] we will administer Patient-Reported Outcomes Measurement Information System – Global Health (PROMIS – Global Health), which contains two domains, mental health (Cronbach's α 0.86) and physical health (Cronbach's α 0.81).[123]

11.3. Mao Expectancy of Treatment Effects (METE)

Others and we have found that outcome expectancy is associated with treatment responses.[124-126] Thus, we will explore whether expectancy is associated with treatment outcomes in the MELODY study**.** METE is a four-item instrument originally developed as the Acupuncture Expectancy Scale by Dr. Mao (PI) to measure outcome expectancy. It has demonstrated reliability (Cronbach's α of 0.82) and validity and is positively correlated with patient self-reported efficacy and satisfaction.[124,127,128] The score ranges from 4 to 20, with higher scores indicating greater expectancy of benefit. We will administer METE at the baseline assessment (week 0) and at the end of treatment (week 8).

11.4 Treatment Preference

We will assess patient preference for MT versus CBT, as we have done in our prior research.[129] This scale will be administered prior to randomization (week 0) to assess baseline patient preferences based on pre-existing attitudes/beliefs about the two interventions and/or prior experiences with the interventions. This scale will also be administered after treatment (week 8) to explore whether patient preferences change after their experiences with either MT or CBT.

11.5. Music Reward

Prior research has demonstrated variability in how people derive reward and pleasure from music, which may affect experiences with MT.[130] Therefore, we will administer the Barcelona Music Reward Questionnaire (BMRQ) at baseline to assess the level of reward associated with music; this instrument has been validated with a Cronbach's α = 0.92.[130]

11.6. Stigma of Receiving Psychological Help

In some cultures, there is stigma associated with mental health needs, which may affect experiences with CBT.[41,42] Therefore, we will administer the Stigma Scale for Receiving Psychological Help (SSRPH) at baseline; this instrument has been validated (Cronbach's α = 0.72) in different cultures to assess the stigma associated with receiving psychological health from mental health providers.[131,132]

Table 1. Summary of Patient-Reported Outcomes.

Primary/Secondary	Name of Outcome	Validated Instrument	Timepoints (Weeks)
Primary	Anxiety	HADS	0, 4, 8, 16, 26
Secondary	Depression	HADS	0, 4, 8, 16, 26
Secondary	Fatigue	BFI	0, 4, 8, 16, 26
Secondary	Insomnia	ISI	0, 4, 8, 16, 26
Secondary	Pain	BPI	0, 4, 8, 16, 26
Secondary	Cognitive Dysfunction	FACT-Cog	0, 4, 8, 16, 26
Secondary	Quality of Life	PROMIS-Global Health	0, 4, 8, 16, 26
Secondary	Expectancy	METE	0, 8

Secondary	Treatment Preference	Treatment Preference Scale	0, 8
Secondary	Music Reward	BMRQ	0
Secondary	Mental Health Stigma	SSRPH	0
Secondary	GAD	GAD-7	0

11.7. Covariates

At the baseline assessment (week 0), we will collect sociodemographics (e.g. age, sex, race/ethnicity, education) and other relevant clinical characteristics (e.g. cancer type, stage, treatment history, time since cancer diagnosis). We will also administer the GAD-7 to screen for generalized anxiety disorder (GAD) at baseline.[133] We will track use of medications prescribed for anxiety (e.g. antidepressants, anxiolytics, hypnotics, sedatives) by asking patients to complete weekly medication diaries at weeks 0, 8, and 26.

11.8. Semi-Structured Interviews.

The impact of musical experiences may be difficult to capture through quantitative measures alone. Methodological experts also recommended mixed methods approaches to understand patient experiences with MT and digital health interventions.[134-136] The qualitative method specialist from MSK's Patient-Reported Outcomes, Community-Engagement and Language (PRO-CEL) Core Facility will conduct 45-minute, semi-structured individual interviews over the phone at week 8 (end of intervention). To reach thematic saturation from our interviews, we will purposively sample N=30 survivors in each treatment arm to be interviewed (total N=60), aiming for balance across sex, race, and treatment response. We chose to conduct interviews immediately after the intervention period to enhance recall of personal experiences during therapy sessions. We will use a semi-structured guide (Appendix C), covering these topics: 1) Acceptability: treatment satisfaction, usefulness of intervention, barriers and facilitators to session attendance, adherence to and engagement with between-session assignments; 2) impact on anxiety symptoms and coping: perceived benefits/harms of intervention on anxiety and co-morbid symptoms, how one copes with stress and anxiety, integration of learned skills into daily life, plans for maintaining anxiety management skills; 3) digital literacy and experience: views towards technology, comfort level with telehealth, transfer of acquired digital skills to other aspects of daily living; and 4) unexpected experiences: unanticipated benefits, harms, or insights from engaging with the interventions. The interview guide will include the flexibility to ask probing questions to enable participants to elaborate on their experiences. All interviews will be audio-recorded and transcribed for thematic analysis.

12.0 CRITERIA FOR REMOVAL FROM STUDY

Any subjects experiencing a serious adverse event (SAE) that is thought to be related to the study intervention will be removed from receiving further treatments. Patients also will be removed from receiving further treatment if they miss two consecutive visits without notification of study staff, or if discontinuation from the treatment is deemed by the PI to be in

their best interest. Subjects discontinued from the treatment aspects of the clinical trial will be scheduled for the Week 4, 8, 16, and 26 evaluations and given appropriate treatment referrals. For the Week 4, 8, 16, and 26 visits, subjects will receive all assessments that were scheduled for these study visits. Any subject withdrawing their consent to participate in the study or their authorization to use their protected health information will be withdrawn from the study. Subjects will be informed during the consent discussion that treatment may be discontinued due to: 1) Intolerable side effects (side effects felt by the patient, therapist, or physician to be of greater severity than the potential benefit from treatment); or 2) failure to attend 2 consecutive MT or CBT visits without notification of study staff. If patients fail to attend sessions with notification, every effort will be made to reschedule the patient such that they can receive the maximum number of treatments.

Reasons for subject discontinuation from the clinical trial will be documented on the Study Termination Form, along with any referrals that are made. We will make every effort to continue to collect data on every subject for the entire study duration regardless of whether or not the subject continues to adhere to the study interventions, assuming the subject has not withdrawn his/her authorization to obtain such information.

13.0 CRITERIA FOR OUTCOME ASSESSMENT AND ENDPOINT EVALUABILITY

13.1 Criteria for Therapeutic Response/Outcome Assessment

MT will be deemed to produce a therapeutic response if it demonstrates non-inferiority to CBT for anxiety symptoms at either 8 weeks or 26 weeks. Our two primary endpoints are HADS anxiety score changes at 8 weeks (end of treatment) and 26 weeks (long-term follow-up). Our rationale for choosing these two assessments for our primary endpoint is as follows. We chose to assess anxiety score changes at 8 weeks to understand the effects of the interventions after patients complete a treatment course. We planned for long-term follow-up of 26 weeks because MT and CBT involve long-term practice of self-management skills. There is also significant interest among cancer survivors in durability of treatment effects.

Based on the linear mixed-effects model coefficients for the time-by-arm interaction at week 8 and week 26, we will perform two tests of whether MT is non-inferior to CBT at reducing HADS anxiety subscale scores within a non-inferiority margin of 0.35 SD for both comparisons. Non-inferiority of MT to CBT will be tested at significance threshold of $p<0.025$ for each comparison, controlling the overall Type I error at 0.05 for our primary endpoint comparisons. We will consider MT to be a promising alternative to CBT for anxiety symptoms if MT is found to be non-inferior to CBT at either week 8 or week 26. Patients and health care providers can use both the short-term (8 week) and long-term (26 week) comparative effectiveness data to inform treatment decision. Please see Section 14.2 for additional details on the analyses.

13.2 Criteria for Study Endpoint Evaluability

Our assessment of the outcomes will be guided by intention-to-treat (ITT) principles, with all patients analyzed in their randomized groups, irrespective of actual treatment received or

number of study assessments completed. All randomized patients will have baseline outcome data and be evaluable for the comparative effectiveness analyses.

14.0 BIOSTATISTICS

This study is a two-arm, parallel group, randomized clinical trial (RCT) to compare the effectiveness of MT versus CBT for anxiety and co-morbid symptoms in a diverse, heterogeneous sample of 300 cancer survivors. Participants will be randomized in a 1:1 ratio (MT: CBT) using randomly permuted blocks of random length, stratified by current anxiety medication use (yes/no), preferred language (English/Spanish), and study site (MSK/MCI) (see Section 7.2). We plan to enroll 300 survivors over a two-year period.

14.1 Populations for Analyses

Consistent with ITT principles, all patients will be analyzed in their randomized groups, irrespective of actual treatment received or number of study assessments completed. All randomized patients will have at least the baseline outcome assessment and will be included in the comparative effectiveness analyses.

14.2 Statistical Analyses.

We describe the analysis for each aim below using the intention-to-treat (ITT) principle (i.e. participants will be analyzed according to their randomly assigned treatment group regardless of drop-out or treatment adherence status). For all specific aims, our main analytic tool will be linear mixed-effects models (LMMs) because our primary outcome (anxiety) and secondary outcomes (e.g. fatigue, insomnia) are repeated continuous outcomes over time.[137] This statistical procedure takes into account within-subject correlations from repeated measurements in the same subjects and allows estimation of between-group differences without necessitating exclusion of participants with missing data. The general template of each LMM will model the outcome as a function of treatment arm and assessment time (categorical), controlling for the randomization stratification variables (baseline anxiety medication use, preferred language, study site), and including a subject-specific random intercept. We will tailor this general LMM template to test the specific aim hypotheses by adding interaction terms (e.g. time-by-intervention) and additional covariates of interest to the model, and by reparametrizing the assessment time variable to focus on specific contrasts.

Aim 1 – Hypotheses 1(a) and 1(b): We will plot the outcome measure trajectories by randomization arm over time and summarize each outcome measure at each assessment time by treatment arm using descriptive statistics. Comparisons between randomization arms with respect to changes in symptom outcomes will be based on specific coefficients from time-by-arm interactions added to the general LMM template described above. Specifically, the model will include all time points (categorical) at which the outcome was assessed (baseline, week 4, 8, 16, 26), and the time-by-arm interaction will include multiple coefficients corresponding to the time-by-arm interaction at each discrete post-baseline time point. Our

statistical inferences, however, will focus on the interaction coefficients at 8 and 26 weeks, which are interpreted as the differences between arms in change from baseline to 8 and 26 weeks, respectively. For Hypothesis 1(a), our two primary endpoints are HADS anxiety score changes at 8 weeks and 26 weeks. Based on the LMM coefficients for the time-by-arm interaction at week 8 and week 26, we will perform two tests of whether MT is non-inferior to CBT at reducing HADS Anxiety within a non-inferiority margin of 0.35 SD for both comparisons. Non-inferiority of MT to CBT will be tested at significance threshold of $p<0.025$ for each comparison, controlling the overall Type I error at 0.05 for our primary endpoint comparisons. For Hypothesis 1(b), we will use a similar LMM evaluating BFI scores over time to test whether the MT arm had significantly greater improvement in fatigue (BFI score) compared to the CBT arm at weeks 8 and 26. In contrast to our primary endpoint non-inferiority comparisons, these tests will be superiority tests. For the other co-morbid symptoms (e.g. depression, insomnia) and quality of life, we will compare the arms using the same methods described for Hypothesis 1(b). Given that a small number of cancer survivors may experience recurrence of disease during the 26-week study, we will conduct sensitivity analyses, excluding those individuals who experienced a recurrence during the study period. Our sensitivity analyses will not replace the primary analysis.

Aim 2 – Heterogeneity of Treatment Effect (HTE): An essential part of patient-centered care is recognizing that not all patients will respond to treatments the same way. We will conduct exploratory, hypothesis-generating HTE analyses to identify patient-level factors associated with treatment response to either MT or CBT by incorporating six relevant variables (i.e. sex, race, ethnicity, education, outcome expectancy, and time since cancer diagnosis) and variable-by-intervention interaction terms in our mixed-effects model described above. For these five exploratory LMM-based analyses, we will guard against inflated Type I error due to multiple testing by adjusting the variable-by-intervention interaction p-values for the false discovery rate.[138,139] We recognize that this type of sub-group analysis, although patient-centered, may need to be interpreted with caution and cannot replace the primary analyses. We intend to approach the evaluation of HTE based on existing literature and patient/clinician input. Our current focus on evaluating and reporting HTE will be based on the approach proposed by Kent et al.[140] However, recognizing other factors may also contribute to treatment responses, we will apply promising emerging Bayesian[141,142] and machine learning[143,144] methods, which can identify HTE and subgroups based on multiple variables simultaneously and are potentially more powerful than traditional univariate methods.

Qualitative Analyses: All interviews will be transcribed verbatim and imported into NVivo Pro version 12.0 for analysis. The semi-structured interviews will be analyzed using theoretical thematic analysis procedures per guidelines by Braun and Clarke.[145] Such analysis is aimed at identifying patterns driven by an *a priori* theoretical framework or research questions. Themes will be identified using a semantic approach in which themes are derived from "the explicit meaning of the data and the analyst is not looking for anything beyond what a participant has said."[145] Two coders will independently code each interview

transcript. Coding discrepancies will be resolved through discussion until consensus is reached. The codes will then be organized into categories, reviewed, and subsequentially organized into broader themes. Interviews from each treatment arm will be analyzed separately, and then compared for convergence and divergence. The themes will be linked to PROs to explore whether specific themes may vary by the magnitude of change in the HADS anxiety score. To augment the HTE analyses, we will also explore whether qualitative themes differed by subgroups (e.g. sex, race, ethnicity, education, high/low expectation) within each treatment group. We will include patient co-investigators during data analyses and interpretation to better understand what types of patient experiences may be unique to MT or CBT. This patient-centered perspective will be critical during the dissemination of study findings to patients and clinicians.

Missing Data: As the only certain way to avoid biases from missing data is to collect complete data,[146] we will minimize the occurrence of missing observations by using a well-piloted clinical trial design,[114] well-trained research staff, and acceptable participant burden with remote assessments that do not require in-person visits. Our prior PCORI trial had only 8% missing data at week 20.[114] By using multiple data collection time points throughout the trial, we will be able to collect patient-reported outcomes and engage patients on a regular basis to help retain patients throughout the 26-week study. Additionally, for patients who have time constraints regarding completing the outcome assessments, we will ask them to only complete the HADS scale (<5 minutes required in our experience). We will ask those who withdraw from the treatment interventions to continue to provide data and we will reimburse them for completing the evaluation. Lastly, for those who voluntarily withdraw from the study, we will record their reasons for withdrawing. Missing data is inevitable in a prospective study, so our second line of defense is to perform sensitivity analyses (e.g. assess impact on results of adjusting for patient disease progression or death) and apply data analysis strategies that are as robust as possible to data losses. We will first explore whether missingness is associated with observed variables (e.g. randomization arm, baseline outcome measures) by comparing patients with complete and incomplete data. Of note, the LMMs described above validly include patients with incomplete data under the missing at random assumption. However, our exploration of the data may deem the missing at random assumption to be inappropriate. In this case, multiple imputation and pattern mixture models are well-established methods we will use to help us deal with these issues.[147,148] We will perform sensitivity analyses to evaluate the robustness of our LMM results by refitting the models after imputing the missing week 8 and week 26 outcomes using multiple imputation. Session recording will be optional for Miami Cancer Institute participants.

Sample Size and Power: Our sample size is based on our primary hypothesis that MT is non-inferior to CBT for anxiety reduction at week 8 and week 26. We will perform two tests of whether MT is non-inferior to CBT at reducing HADS Anxiety at week 8 and also at week 26, each with a non-inferiority margin of D=0.35 SD for both comparisons. These comparisons will be based on LMM coefficients for the time-by-arm interaction at week 8 and week 26;

however, we present our power calculation based on two-sample t-tests of differences between the arms in their change scores. Therefore, our power estimate is conservative; that is, in our LMM-based analysis, we will have slightly higher power than presented below, with all other assumptions held constant. Assuming 150 participants are randomized to each arm, 15% attrition, and a one-sided significance threshold of 0.025 (controlling the overall Type I error at 0.05 for our two primary endpoint comparisons), we will have 80% power to find MT non-inferior to CBT with respect to week 8 and 26 HADS Anxiety scores within a non-inferiority margin of D=0.35 SD. Justification of Non-inferiority Margin: A recent large study used multiple rigorous methods to empirically estimate a difference of 1.7 points as the MCID for the HADS scales.[117] In preliminary data of CBT recipients with baseline HADS Anxiety scores ≥8, the SD for the HADS Anxiety score was 4.2 at week 8 (end of treatment).[114] Additionally, in a large, recently completed acupuncture trial of cancer survivors, among 159 patients with baseline HADS Anxiety scores ≥8, the HADS Anxiety score SD was 4.0 during follow-up.[149,150] Based on these studies, we assume our HADS Anxiety score SD will be approximately 4.2. Dividing the raw-score MCID of 1.7 points by 4.2 yields standardized difference (i.e. Cohen's D) of 0.40. Our non-inferiority margin of D=0.35 SD is smaller than the HADS Anxiety standardized MCID (D=0.40); as such, our non-inferiority margin and statistical approach will find MT to be non-inferior to CBT only if MT is not meaningfully worse than CBT. We confirmed with our patient advisory groups, using concrete examples, that the non-inferiority margin of D=0.35 SD would not represent a meaningful difference between the treatments. Qualitative Analyses: The number of participants required to draw meaningful conclusions from semi-structured interviews is determined by saturation, i.e. the point at which existing themes are fully understood and no new themes emerge through further data collection.[151] Prior research has indicated that thematic saturation can typically be obtained with fewer than 15 interview participants, although these findings were based on relatively homogenous samples.[152] Given that we are seeking to capture perspectives from a diverse group of survivors, we will plan for 30 interviews in each treatment arm to achieve thematic saturation. We achieved saturation at N=30 interviews in an ongoing qualitative study of our virtual integrative medicine program.[153]

15.0 TOXICITIES/RISKS/SIDE EFFECTS

MT and CBT are non-pharmacological interventions. All potential risks that might occur as a result of participation will be detailed in an informed consent form and will also be fully discussed with each patient prior to enrollment. We will also explain to each patient that in the unlikely event of an injury directly resulting from the research procedures, every effort will be made to make the facilities and professional skills of MSK available to them.

Physical Risks: MT and CBT are not associated with any physical risks.

Psychological Risks: During MT and CBT sessions, cancer survivors may experience psychological discomfort when thinking about their experiences with cancer and how it has affected their lives. In addition, study assessments will consist of questions related to socio-demographics, medical history (including cancer type and treatment), anxiety, depression,

fatigue, insomnia, pain, cognitive difficulties, and health-related quality of life. It is possible that participants may feel uncomfortable with some of these questions. Although there is the potential for psychological distress while completing study questionnaires or participating in semi-structured interviews, this has been found to be a rare event in our previous and ongoing studies. CTCAE Version 5 will be utilized for side effect evaluation.

Financial and Legal Risks: There are no financial or legal risks to the study participants. All research interventions and evaluations are provided free of charge to study participants.

Privacy and/or Confidentiality Risks: There is a small risk of loss of privacy or confidentiality as someone could get access to the personal information in the study participants' study records.

15.1 Serious Adverse Event (SAE) Reporting

An adverse event is considered serious if it results in ANY of the following outcomes:

- Death
- A life-threatening adverse event
- An adverse event that results in inpatient hospitalization or prolongation of existing hospitalization
- A persistent or significant incapacity or substantial disruption of the ability to conduct normal life functions
- A congenital anomaly/birth defect
- Important Medical Events (IME) that may not result in death, be life threatening, or require hospitalization may be considered serious when, based upon medical judgment, they may jeopardize the patient or participant and may require medical or surgical intervention to prevent one of the outcomes listed in this definition
- *List any additional events that require SAE reporting (pregnancy, AEs of special interest (AESI), secondary malignancies, etc.)*

Note: Hospital admission for a planned procedure/disease treatment is not considered an SAE.

SAE reporting is required as soon as the participant starts investigational treatment/intervention. SAE reporting is required for 30 days after the participant's last investigational treatment/intervention. Any event that occurs after the 30-day period that is unexpected and at least possibly related to protocol treatment must be reported.

Please note: Any SAE that occurs prior to the start of investigational treatment/intervention and is related to a screening test or procedure (e.g., a screening biopsy) must be reported.

All SAEs must be submitted in PIMS. If an SAE requires submission to the HRPP Office per IRB SOP RR-408 'Reporting of Serious Adverse Events,' the SAE report must be submitted

within 5 calendar days of the event. All other SAEs must be submitted within 30 calendar days of the event.

The report should contain the following information:

- The date the adverse event occurred
- The adverse event
- The grade of the event
- Relationship of the adverse event to the treatment(s)
- If the AE was expected
- Detailed text that includes the following
    - An explanation of how the AE was handled
    - A description of the participant's condition
    - Indication if the participant remains on the study
- If an amendment will need to be made to the protocol and/or consent form
- If the SAE is an Unanticipated Problem

16.0 PROTECTION OF HUMAN PARTICIPANTS

Voluntary Nature of the Study: All study participants will provide informed consent prior to enrolling. The participant will be informed of the procedures to be followed, alternatives, potential benefits, side effects, risks, and discomforts. We will inform patients that they are free to withdraw from the study at any time and that their participation is voluntary.

Payments to Participants: Participants will receive $40 after completing the questionnaire at week 8 and $60 after completing the questionnaire at week 26.

Protection Against Risk: The PI has led two NIH-funded trials of major depressive disorder and generalized anxiety disorder. He has both clinical and research experience in mental health diagnosis and treatment. During study assessments, our well-trained research staff will be readily available by phone/email/videoconference to assist patients as needed, and our qualitative methods specialist has extensive experience in conducting interviews on potentially sensitive topics in a safe and appropriate manner. If a research team member observes a study participant displaying elevated psychological distress, they will inform the study clinicians so that they can evaluate and potentially refer the patient to MSK's psychiatry services, if clinically warranted. In addition, all study therapists must undergo a standardized training process and meet a rigorous certification standard before delivering any treatments to research participants. All therapists are specifically trained to build rapport, develop a psychotherapeutic relationship, and establish a supportive context. Treatment fidelity is routinely monitored and audited to ensure that therapists are following the protocol and adhering to safety procedures. Every effort will be made to ensure the safety and comfort of the study subjects. The therapists will monitor the patients for adverse events during each session, and report any potential AEs to the PI. In the unlikely event of significant

psychological distress, we will refer participants for urgent evaluation by MSK's Psychiatry Service. If a patient displays active suicidal ideation and poses a significant and acute risk of self-harm, we will contact the patient immediately to connect the patient with emergency psychiatric assessment and care.

Alternative Treatment: The alternative to participating in the study is to receive other non-pharmacologic interventions (e.g. other forms of psychotherapy besides CBT) or pharmacologic agents (e.g. anxiolytics, antidepressants). During the informed consent process, potential study subjects will be informed that these alternative treatments are available, that their participation in the study is entirely voluntary, and that their care will not be affected in any way if they decide not to participate in the study.

Risk/Benefits Ratio: With the American cancer survivor population expected to exceed 22 million by 2030, there is a pressing need to address anxiety, an exceedingly common long-term consequence of cancer, in this growing population. The COVID-19 pandemic has not only exacerbated psychological distress, but also highlighted many of the pre-existing barriers to mental health resources and services. If successful, our proposed research on virtually delivered MT and CBT has potential to address some of these barriers and allow more patients to access evidence-based interventions for anxiety. The risks to subjects are small in comparison to the valuable and timely scientific information and potential clinical benefits that will result from the conduct of this study.

16.1 Privacy

MSK's Privacy Office may allow the use and disclosure of protected health information pursuant to a completed and signed Research Authorization form. The use and disclosure of protected health information will be limited to the individuals/entities described in the Research Authorization form. A Research Authorization form must be approved by the IRB and Privacy Board (IRB/PB).

The consent indicates that individualized, de-identified information collected for the purposes of this study may be shared with other qualified researchers. Only researchers who have received approval from MSK will be allowed to access this information, which will not include protected health information such as the participant's name, except for dates. It is also stated in the Research Authorization that their research data may be shared with others at the time of study publication.

16.2 Data Management

The CRC assigned to this study will be responsible for project compliance, data collection, abstraction and entry, data reporting, regulatory and quality control monitoring, problem identification, and prioritization. Coordination of the study team activities will be the responsibility of our Clinical Research Supervisor (CRS) and/or Clinical Research Manager (CRM). The CRS and CRM will work with the CRC on problem resolution, organization, and quality control. The PI will hold weekly meetings with the research staff to review study

progress and to manage any issues that arise. For any communication with participants, all security precautions will be taken. The data collected for this study will be entered secure databases (i.e. CRDB, Excel, or REDCap [Research Electronic Data Capture], based on the database functionality. A minimal dataset will be entered into CRDB, and a study tracker will be in Excel. Participants will be asked to complete patient reported outcomes assessments online using REDCap. If they prefer, patients will have the option to complete the measures via pencil and paper on scannable forms or over the phone with a CRC to reduce participant burden and ensure timely completion.

REDCap is a data management software system supported by the Clinical Research Administration (CRA) at MSK. Members of the CRA supporting the REDCap software will have access to REDCap projects hosted by MSK‟s servers for the purpose of ensuring the proper functioning of the database and the overall software system. REDCap is a tool for the creation of customized, secure data management systems including web-based data entry forms, reporting tools, and a full array of security features including user- and group-based privileges with a full audit trail of data manipulation an export procedures. REDCap is maintained on MSK-owned servers that are kept in a locked server room with appropriate environmental modifications (e.g. proper ventilation, power redundancy and fault tolerance arrangement) and backed up nightly with some back-up tapes stored off-site. The MSK Information Systems group is responsible for applying all operating system patches and security updates to the REDCap servers. All connections to REDCap utilize encrypted (SSL-based) connections. Nationally, the REDCap software is developed, enhanced, and supported through a multi-institutional consortium led by Vanderbilt University.

Source documentation will be available to support the computerized patient data. The confidentiality of patient information will be carefully protected. Following data entry by Integrative Medicine Service research staff, data will be maintained in a secure location in the Integrative Medicine offices.

Final data sets for publication are required to be locked and stored centrally for potential future access requests from outside entities.

16.3 Quality Assurance

Weekly registration reports will be generated to monitor patient accruals and completeness of registration data. Routine data quality reports will be generated to assess missing data and inconsistencies. Accrual rates and extent and accuracy of evaluations and follow-up will be monitored periodically throughout the study period and potential problems will be brought to the attention of the study team for discussion and action. Random-sample data quality and protocol compliance audits will be conducted by the study team at a minimum of two times per year and more frequently if indicated.

16.4 Data and Safety Monitoring

The Data and Safety Monitoring Plan utilized for this study must align with the MSK DSM Plan where applicable.

The Data and Safety Monitoring (DSM) Plans at Memorial Sloan Kettering were approved by the National Cancer Institute in August 2018. The plans address the new policies set forth by the NCI in the document entitled "Policy of the National Cancer Institute for Data and Safety Monitoring of Clinical Trials."

There are several different mechanisms by which clinical studies are monitored for data safety and quality. At a departmental/PI level, there exist procedures for quality control by the research team(s). Institutional processes in place for quality assurance include protocol monitoring, compliance and data verification audits, staff education on clinical research QA, and two institutional committees that are responsible for monitoring the activities of our clinical trials programs. The committees: *Data and Safety Monitoring Committee (DSMC)* for Phase I and II clinical trials, and the *Data and Safety Monitoring Board (DSMB)* for Phase III clinical trials, report to the Deputy Physician-in-Chief of Clinical Research.

The degree of monitoring required will be determined based on level of risk and documented.

The MSK DSMB monitors phase III trials and the DSMC monitors non-phase III trials. The DSMB/C have oversight over the following trials:

- MSK Investigator-Initiated Trials (IITs; MSK as sponsor)
- External studies where MSK is the data coordinating center
- Low risk studies identified as requiring DSMB/C review

The DSMC will initiate review following the enrollment of the first participant, or by the end of the year one if no accruals, and will continue for the study lifecycle until there are no participants under active therapy and the protocol has closed to accrual. The DSMB will initiate review once the protocol is open to accrual.

17.0 REFERENCES

1. Miller KD, Nogueira L, Mariotto AB, et al. Cancer treatment and survivorship statistics, 2019. *CA: a cancer journal for clinicians.* 2019;69(5):363-385.
2. Beekers N, Husson O, Mols F, van Eenbergen M, van de Poll-Franse LV. Symptoms of Anxiety and Depression Are Associated With Satisfaction With Information Provision and Internet Use Among 3080 Cancer Survivors: Results of the PROFILES Registry. *Cancer nursing.* 2014.
3. Greer JA, Solis JM, Temel JS, et al. Anxiety disorders in long-term survivors of adult cancers. *Psychosomatics.* 2011;52(5):417-423.
4. Boyes AW, Girgis A, D'Este C, Zucca AC. Flourishing or floundering? Prevalence and correlates of anxiety and depression among a population-based sample of adult cancer survivors 6months after diagnosis. *J Affect Disord.* 2011;135(1-3):184-192.
5. Boyes AW, Girgis A, D'Este CA, Zucca AC, Lecathelinais C, Carey ML. Prevalence and predictors of the short-term trajectory of anxiety and depression in the first year after a

cancer diagnosis: a population-based longitudinal study. *Journal of clinical oncology : official journal of the American Society of Clinical Oncology.* 2013;31(21):2724-2729.

6. Maass SW, Roorda C, Berendsen AJ, Verhaak PF, de Bock GH. The prevalence of long-term symptoms of depression and anxiety after breast cancer treatment: A systematic review. *Maturitas.* 2015.
7. Mehnert A, Koch U. Psychological comorbidity and health-related quality of life and its association with awareness, utilization, and need for psychosocial support in a cancer register-based sample of long-term breast cancer survivors. *Journal of psychosomatic research.* 2008;64(4):383-391.
8. Faller H, Strahl A, Richard M, Niehues C, Meng K. Symptoms of depression and anxiety as predictors of physical functioning in breast cancer patients. A prospective study using path analysis. *Acta oncologica (Stockholm, Sweden).* 2017;56(12):1677-1681.
9. Oberoi DV, White VM, Seymour JF, et al. Distress and unmet needs during treatment and quality of life in early cancer survivorship: A longitudinal study of haematological cancer patients. *European journal of haematology.* 2017;99(5):423-430.
10. Oerlemans S, Mols F, Nijziel MR, Zijlstra WP, Coebergh JW, van de Poll-Franse LV. The course of anxiety and depression for patients with Hodgkin's lymphoma or diffuse large B cell lymphoma: a longitudinal study of the PROFILES registry. *Journal of cancer survivorship : research and practice.* 2014;8(4):555-564.
11. Lidgren M, Wilking N, Jönsson B, Rehnberg C. Health related quality of life in different states of breast cancer. *J Quality of Life Research.* 2007;16(6):1073-1081.
12. Grov EK, Fosså SD, Dahl AA. Insomnia in elderly cancer survivors--a population-based controlled study of associations with lifestyle, morbidity, and psychosocial factors. Results from the Health Survey of North-Trøndelag County (HUNT-2). Insomnia in elderly cancer survivors. *Supportive care in cancer : official journal of the Multinational Association of Supportive Care in Cancer.* 2011;19(9):1319-1326.
13. Brintzenhofe-Szoc KM, Levin TT, Li Y, Kissane DW, Zabora JR. Mixed anxiety/depression symptoms in a large cancer cohort: prevalence by cancer type. *Psychosomatics.* 2009;50(4):383-391.
14. Gold M, Dunn LB, Phoenix B, et al. Co-occurrence of anxiety and depressive symptoms following breast cancer surgery and its impact on quality of life. *European journal of oncology nursing : the official journal of European Oncology Nursing Society.* 2016;20:97-105.
15. Shim EJ, Jeong D, Moon HG, et al. Profiles of depressive symptoms and the association with anxiety and quality of life in breast cancer survivors: a latent profile analysis. *Quality of life research : an international journal of quality of life aspects of treatment, care and rehabilitation.* 2020;29(2):421-429.
16. Stone P, Richards M, A'Hern R, Hardy J. Fatigue in patients with cancers of the breast or prostate undergoing radical radiotherapy. *Journal of pain and symptom management.* 2001;22(6):1007-1015.
17. Geinitz H, Zimmermann FB, Thamm R, Keller M, Busch R, Molls M. Fatigue in patients with adjuvant radiation therapy for breast cancer: long-term follow-up. *Journal of cancer research and clinical oncology.* 2004;130(6):327-333.
18. Dhruva A, Dodd M, Paul SM, et al. Trajectories of fatigue in patients with breast cancer before, during, and after radiation therapy. *Cancer nursing.* 2010;33(3):201-212.
19. Reinertsen KV, Cvancarova M, Loge JH, Edvardsen H, Wist E, Fosså SD. Predictors and course of chronic fatigue in long-term breast cancer survivors. *Journal of cancer survivorship : research and practice.* 2010;4(4):405-414.
20. Bushnell MC, Ceko M, Low LA. Cognitive and emotional control of pain and its disruption in chronic pain. *Nat Rev Neurosci.* 2013;14(7):502-511.

21. Ferreri F, Lapp LK, Peretti CS. Current research on cognitive aspects of anxiety disorders. *Current opinion in psychiatry.* 2011;24(1):49-54.
22. Babcock ZR, Kogut SJ, Vyas A. Association between polypharmacy and health-related quality of life among cancer survivors in the United States. *Journal of cancer survivorship : research and practice.* 2020;14(1):89-99.
23. Keats MR, Cui Y, DeClercq V, Grandy SA, Sweeney E, Dummer TJB. Burden of multimorbidity and polypharmacy among cancer survivors: a population-based nested case-control study. *Supportive care in cancer : official journal of the Multinational Association of Supportive Care in Cancer.* 2020.
24. Murphy CC, Fullington HM, Alvarez CA, et al. Polypharmacy and patterns of prescription medication use among cancer survivors. *Cancer.* 2018;124(13):2850-2857.
25. Vyas A, Alghaith G, Hufstader-Gabriel M. Psychotropic polypharmacy and its association with health-related quality of life among cancer survivors in the USA: a population-level analysis. *Quality of life research : an international journal of quality of life aspects of treatment, care and rehabilitation.* 2020;29(8):2029-2037.
26. Bradt J, Dileo C, Magill L, Teague A. Music interventions for improving psychological and physical outcomes in cancer patients. *The Cochrane database of systematic reviews.* 2016(8):CD006911.
27. Bro ML, Jespersen KV, Hansen JB, et al. Kind of blue: A systematic review and meta-analysis of music interventions in cancer treatment. *Psychooncology.* 2018;27(2):386-400.
28. Gramaglia C, Gambaro E, Vecchi C, et al. Outcomes of music therapy interventions in cancer patients-A review of the literature. *Crit Rev Oncol Hematol.* 2019;138:241-254.
29. Köhler F, Martin ZS, Hertrampf RS, et al. Music Therapy in the Psychosocial Treatment of Adult Cancer Patients: A Systematic Review and Meta-Analysis. *Frontiers in psychology.* 2020;11:651.
30. Osborn RL, Demoncada AC, Feuerstein M. Psychosocial interventions for depression, anxiety, and quality of life in cancer survivors: meta-analyses. *Int J Psychiatry Med.* 2006;36(1):13-34.
31. Moyer A, Sohl SJ, Knapp-Oliver SK, Schneider S. Characteristics and methodological quality of 25 years of research investigating psychosocial interventions for cancer patients. *Cancer treatment reviews.* 2009;35(5):475-484.
32. Ye M, Du K, Zhou J, et al. A meta-analysis of the efficacy of cognitive behavior therapy on quality of life and psychological health of breast cancer survivors and patients. *Psychooncology.* 2018;27(7):1695-1703.
33. Butler AC, Chapman JE, Forman EM, Beck AT. The empirical status of cognitive-behavioral therapy: a review of meta-analyses. *Clinical psychology review.* 2006;26(1):17-31.
34. Otto MW, Smits JA, Reese HE. Cognitive-behavioral therapy for the treatment of anxiety disorders. *The Journal of clinical psychiatry.* 2004;65 Suppl 5:34-41.
35. Olatunji BO, Cisler JM, Deacon BJ. Efficacy of cognitive behavioral therapy for anxiety disorders: a review of meta-analytic findings. *The Psychiatric clinics of North America.* 2010;33(3):557-577.
36. Andersen BL, DeRubeis RJ, Berman BS, et al. Screening, assessment, and care of anxiety and depressive symptoms in adults with cancer: an American Society of Clinical Oncology guideline adaptation. *Journal of clinical oncology : official journal of the American Society of Clinical Oncology.* 2014;32(15):1605-1619.
37. Lyman GH, Greenlee H, Bohlke K, et al. Integrative Therapies During and After Breast Cancer Treatment: ASCO Endorsement of the SIO Clinical Practice Guideline. *Journal of clinical oncology : official journal of the American Society of Clinical Oncology.* 2018;36(25):2647-2655.

38. National Comprehensive Cancer Network. NCCN Clinical Practice Guidelines in Oncology: Distress Management. Version 1.2021 - September 30, 2020. <https://www.nccn.org/professionals/physician_gls/pdf/distress.pdf> Accessed December 1, 2020.
39. Edmonds M, Hadjistavropoulos HD, Schneider LH, Dear BF, Titov N. Who benefits most from therapist-assisted internet-delivered cognitive behaviour therapy in clinical practice? Predictors of symptom change and dropout. *Journal of anxiety disorders.* 2018;54:24-32.
40. Beatty L, Binnion C. A Systematic Review of Predictors of, and Reasons for, Adherence to Online Psychological Interventions. *International journal of behavioral medicine.* 2016;23(6):776-794.
41. Eylem O, de Wit L, van Straten A, et al. Stigma for common mental disorders in racial minorities and majorities a systematic review and meta-analysis. *BMC public health.* 2020;20(1):879.
42. McGuire TG, Miranda J. New evidence regarding racial and ethnic disparities in mental health: policy implications. *Health affairs (Project Hope).* 2008;27(2):393-403.
43. Greenlee H, Balneaves LG, Carlson LE, et al. Clinical practice guidelines on the use of integrative therapies as supportive care in patients treated for breast cancer. *Journal of the National Cancer Institute Monographs.* 2014;2014(50):346-358.
44. Greenlee H, DuPont-Reyes MJ, Balneaves LG, et al. Clinical practice guidelines on the evidence-based use of integrative therapies during and after breast cancer treatment. *CA: a cancer journal for clinicians.* 2017;67(3):194-232.
45. Atkinson TM, Liou KT, Borten MA, et al. Association Between Music Therapy Techniques and Patient-Reported Moderate to Severe Fatigue in Hospitalized Adults With Cancer. *JCO oncology practice.* 2020:Op2000096.
46. Wang Y, Duan Z, Ma Z, et al. Epidemiology of mental health problems among patients with cancer during COVID-19 pandemic. *Translational psychiatry.* 2020;10(1):263.
47. Ramaswamy A, Yu M, Drangsholt S, et al. Patient Satisfaction With Telemedicine During the COVID-19 Pandemic: A Retrospective Cohort Study. *Journal of medical Internet research.* 2020.
48. Institute of Medicine Committee on Psychosocial Services to Cancer Patients/Families in a Community S. The National Academies Collection: Reports funded by National Institutes of Health. In: Adler NE, Page AEK, eds. *Cancer Care for the Whole Patient: Meeting Psychosocial Health Needs.* Washington (DC): National Academies Press (US)

Copyright © 2008, National Academy of Sciences.; 2008.
49. Mitchell AJ, Ferguson DW, Gill J, Paul J, Symonds P. Depression and anxiety in long-term cancer survivors compared with spouses and healthy controls: a systematic review and meta-analysis. *The Lancet Oncology.* 2013;14(8):721-732.
50. Whitney RL, Bell JF, Bold RJ, Joseph JG. Mental health needs and service use in a national sample of adult cancer survivors in the USA: Has psychosocial care improved? *Psycho-Oncology.* 2015;24(1):80-88.
51. Haskins CB, McDowell BD, Carnahan RM, et al. Impact of preexisting mental illness on breast cancer endocrine therapy adherence. *Breast Cancer Research and Treatment.* 2019;174(1):197-208.
52. Smith KL, Yeruva SLH, Blackford A, et al. Predictors of adherence to adjuvant endocrine therapy (ET) for early breast cancer (BC) in a prospective clinic-based cohort. *Cancer Research.* 2018;78(4).
53. Bender CM, Gentry AL, Brufsky AM, et al. Influence of patient and treatment factors on adherence to adjuvant endocrine therapy in breast cancer. *Oncology nursing forum.* 2014;41(3):274-285.

54. Khushalani JS, Qin J, Cyrus J, et al. Systematic review of healthcare costs related to mental health conditions among cancer survivors. *Expert review of pharmacoeconomics & outcomes research.* 2018;18(5):505-517.
55. Harris JP, Kashyap M, Humphreys JN, Pollom EL, Chang DT. The clinical and financial cost of mental disorders among elderly patients with gastrointestinal malignancies. *Cancer medicine.* 2020.
56. Barlow DH. *Anxiety and its disorders: The nature and treatment of anxiety and panic, 2nd ed.* New York, NY, US: Guilford Press; 2002.
57. Freeman A. *Clinical applications of cognitive therapy.* New York: Kluwer Academic/Plenum Publishers; 2004.
58. Given CW. *Evidence-based cancer care and prevention : behavioral interventions.* New York, NY: Springer Pub. Co.; 2003.
59. Moorey SG, S. *Cognitive behaviour therapy for people with cancer.* New York: Oxford University Press; 2002.
60. Greer JA, Park ER, Prigerson HG, Safren SA. Tailoring Cognitive-Behavioral Therapy to Treat Anxiety Comorbid with Advanced Cancer. *J Cogn Psychother.* 2010;24(4):294-313.
61. Sun H, Huang H, Ji S, et al. The Efficacy of Cognitive Behavioral Therapy to Treat Depression and Anxiety and Improve Quality of Life Among Early-Stage Breast Cancer Patients. *Integrative cancer therapies.* 2019;18:1534735419829573.
62. Getu MA, Chen C, Panpan W, Mboineki JF, Dhakal K, Du R. The effect of cognitive behavioral therapy on the quality of life of breast cancer patients: a systematic review and meta-analysis of randomized controlled trials. *Quality of life research : an international journal of quality of life aspects of treatment, care and rehabilitation.* 2020.
63. Andrews G, Cuijpers P, Craske MG, McEvoy P, Titov N. Computer therapy for the anxiety and depressive disorders is effective, acceptable and practical health care: a meta-analysis. *PloS one.* 2010;5(10):e13196.
64. Hadjistavropoulos HD, Nugent MM, Alberts NM, Staples L, Dear BF, Titov N. Transdiagnostic Internet-delivered cognitive behaviour therapy in Canada: An open trial comparing results of a specialized online clinic and nonspecialized community clinics. *Journal of anxiety disorders.* 2016;42:19-29.
65. O'Callaghan C, Magill L. Music therapy with adults diagnosed with cancer and their families. *Oxford handbook of music therapy.* 2016:112-134.
66. Lepore SJ. A social-cognitive processing model of emotional adjustment to cancer. In: Baum A, Anderson B, eds. *Psychosocial interventions for cancer.* Washington, D.C.: American Psychological Association; 2001:99-116.
67. Lepore SJ, Helgeson VS. Social constraints, intrusive thoughts, and mental health after prostate cancer. *Journal of Social and Clinical Psychology.* 1998;17(1):89-106.
68. Harper FW, Schmidt JE, Beacham AO, et al. The role of social cognitive processing theory and optimism in positive psychosocial and physical behavior change after cancer diagnosis and treatment. *Psychooncology.* 2007;16(1):79-91.
69. Koelsch S. Brain correlates of music-evoked emotions. *Nat Rev Neurosci.* 2014;15(3):170-180.
70. Overy K, Molnar-Szakacs I. Being Together in Time: Musical Experience and the Mirror Neuron System. *Music Perception.* 2009;26(5):489-504.
71. Hargreaves DJ. *The social psychology of music.* Oxford: Oxford Univ. Press; 2009.
72. Amir D. Giving Trauma a Voice: The Role of Improvisational Music Therapy in Exposing, Dealing with and Healing a Traumatic Experience of Sexual Abuse. *Music Therapy Perspectives.* 2004;22(2):96-103.

73. Bensimon M, Amir D, Wolf Y. Drumming through trauma: Music therapy with post-traumatic soldiers. *The Arts in Psychotherapy.* 2008;35(1):34-48.
74. Koelsch S, Skouras S. Functional centrality of amygdala, striatum and hypothalamus in a "small-world" network underlying joy: an fMRI study with music. *Human brain mapping.* 2014;35(7):3485-3498.
75. Finn S, Fancourt D. The biological impact of listening to music in clinical and nonclinical settings: A systematic review. *Progress in brain research.* 2018;237:173-200.
76. Chanda ML, Levitin DJ. The neurochemistry of music. *Trends in cognitive sciences.* 2013;17(4):179-193.
77. White JM. Effects of relaxing music on cardiac autonomic balance and anxiety after acute myocardial infarction. *American journal of critical care : an official publication, American Association of Critical-Care Nurses.* 1999;8(4):220-230.
78. Koelsch S, Jancke L. Music and the heart. *European heart journal.* 2015;36(44):3043-3049.
79. Bradt J, Dileo C, Potvin N. Music for stress and anxiety reduction in coronary heart disease patients. *The Cochrane database of systematic reviews.* 2013(12):Cd006577.
80. McPherson T, Berger D, Alagapan S, Frohlich F. Active and Passive Rhythmic Music Therapy Interventions Differentially Modulate Sympathetic Autonomic Nervous System Activity. *Journal of music therapy.* 2019;56(3):240-264.
81. Reeves JW, Fisher AJ, Newman MG, Granger DA. Sympathetic and hypothalamic-pituitary-adrenal asymmetry in generalized anxiety disorder. *Psychophysiology.* 2016;53(6):951-957.
82. Holwerda SW, Luehrs RE, Gremaud AL, et al. Relative burst amplitude of muscle sympathetic nerve activity is an indicator of altered sympathetic outflow in chronic anxiety. *Journal of neurophysiology.* 2018;120(1):11-22.
83. Rauch SL, Shin LM, Wright CI. Neuroimaging studies of amygdala function in anxiety disorders. *Annals of the New York Academy of Sciences.* 2003;985:389-410.
84. Tafet GE, Nemeroff CB. Pharmacological Treatment of Anxiety Disorders: The Role of the HPA Axis. *Frontiers in psychiatry.* 2020;11:443.
85. Mehr SA, Singh M, Knox D, et al. Universality and diversity in human song. *Science (New York, NY).* 2019;366(6468).
86. Savage PE, Brown S, Sakai E, Currie TE. Statistical universals reveal the structures and functions of human music. *Proceedings of the National Academy of Sciences of the United States of America.* 2015;112(29):8987-8992.
87. Yun H, Sun L, Mao JJ. Growth of Integrative Medicine at Leading Cancer Centers Between 2009 and 2016: A Systematic Analysis of NCI-Designated Comprehensive Cancer Center Websites. *Journal of the National Cancer Institute Monographs.* 2017;2017(52):29-32.
88. Desai K, Liou KT, Liang K, Seluzicki CM, Mao JJ. Availability of Integrative Medicine Services at National Cancer Institute-Designated Comprehensive Cancer Centers and Community Hospitals. *Under Review.*
89. Internet/Broadband Fact Sheet. 2019; https://www.pewresearch.org/internet/fact-sheet/internet-broadband/#internet-use-over-time. Accessed September 14, 2020.
90. Mobile Technology and Home Broadband 2019; https://www.pewresearch.org/internet/2019/06/13/mobile-technology-and-home-broadband-2019/. Accessed September 14, 2020.
91. Harris J, Cheevers K, Armes J. The emerging role of digital health in monitoring and supporting people living with cancer and the consequences of its treatments. *Current opinion in supportive and palliative care.* 2018;12(3):268-275.
92. Zoom Video Communications Inc (ZM) Q1 2020 Earnings Call Transcript. https://www.fool.com/earnings/call-transcripts/2020/06/03/zoom-video-communications-inc-zm-q1-2021-earnings.aspx. Accessed August 12, 2020.

93. Roberts ET, Mehrotra A. Assessment of Disparities in Digital Access Among Medicare Beneficiaries and Implications for Telemedicine. *JAMA internal medicine.* 2020.
94. Vaudreuil R, Langston DG, Magee WL, Betts D, Kass S, Levy C. Implementing music therapy through telehealth: considerations for military populations. *Disability and rehabilitation Assistive technology.* 2020:1-10.
95. Bronson H, Vaudreuil R, Bradt J. Music Therapy Treatment of Active Duty Military: An Overview of Intensive Outpatient and Longitudinal Care Programs. *Music Therapy Perspectives.* 2018;36(2):195-206.
96. Levy CE, Spooner H, Lee JB, Sonke J, Myers K, Snow E. Telehealth-based creative arts therapy: Transforming mental health and rehabilitation care for rural veterans. *The Arts in Psychotherapy.* 2018;57:20-26.
97. Lightstone AJ, Bailey SK, Voros P. Collaborative music therapy via remote video technology to reduce a veteran's symptoms of severe, chronic PTSD. *Arts & Health.* 2015;7(2):123-136.
98. Spooner H, Lee JB, Langston DG, Sonke J, Myers KJ, Levy CE. Using distance technology to deliver the creative arts therapies to veterans: Case studies in art, dance/movement and music therapy. *The Arts in Psychotherapy.* 2019;62:12-18.
99. Knott D, Block S. Virtual Music Therapy: Developing New Approaches to Service Delivery. *Music Therapy Perspectives.* 2020.
100. Gooding LF, Trainor B. Working with parents in the neonatal intensive care unit: An analysis of music therapy practice in the United States of America. *The Arts in Psychotherapy.* 2018;59:1-6.
101. Magee WL. *Music technology in therapeutic and health settings.* London: Jessica Kingsley Publishers; 2014.
102. American Music Therapy Association. COVID-19 Resources for Music Therapists and Students. Telehealth Considerations and Resources. . https://www.musictherapy.org/about/covid19_resources/#Telehealth%20Considerations%20and%20Resources. Accessed September 2, 2020.
103. Andersson G. Internet-Delivered Psychological Treatments. *Annual review of clinical psychology.* 2016;12:157-179.
104. Olthuis JV, Watt MC, Bailey K, Hayden JA, Stewart SH. Therapist-supported Internet cognitive behavioural therapy for anxiety disorders in adults. *The Cochrane database of systematic reviews.* 2016;3(3):Cd011565.
105. American Psychological Association. Resolution on the Recognition of Psychotherapy Effectiveness. Approved August 2012. <https://www.apa.org/about/policy/resolution-psychotherapy> Accessed on December 15, 2020.
106. Thompson M, Tiwari A, Fu R, Moe E, Buckley DI. AHRQ Methods for Effective Health Care. In: *A Framework To Facilitate the Use of Systematic Reviews and Meta-Analyses in the Design of Primary Research Studies.* Rockville (MD): Agency for Healthcare Research and Quality (US); 2012.
107. Chow S, Wan BA, Pidduck W, et al. Symptoms Predictive of Overall Quality of Life Using the Edmonton Symptom Assessment Scale in Breast Cancer Patients Receiving Radiotherapy. *Clinical breast cancer.* 2019;19(6):405-410.
108. American College of Surgeons Commission on Cancer. Optimal Resources for Cancer Care: 2020 Standards. Chicago, IL. 2020. In.
109. Taruffi L, Pehrs C, Skouras S, Koelsch S. Effects of Sad and Happy Music on Mind-Wandering and the Default Mode Network. *Scientific reports.* 2017;7(1):14396.
110. Bradt J, Potvin N, Kesslick A, et al. The impact of music therapy versus music medicine on psychological outcomes and pain in cancer patients: a mixed methods study. *Supportive*

care in cancer : official journal of the Multinational Association of Supportive Care in Cancer. 2015;23(5):1261-1271.
111. Yates GJ, Silverman MJ. Immediate effects of single-session music therapy on affective state in patients on a post-surgical oncology unit: A randomized effectiveness study. *The Arts in Psychotherapy.* 2015;44:57-61.
112. Trevino KM, Lachs M, Li Y, et al. Managing Anxiety from Cancer (MAC): A pilot randomized controlled trial of an anxiety intervention for older adults with cancer and their caregivers. *Palliative and Supportive Care.* under review.
113. Deshields T, Kracen A, Nanna S, Kimbro L. Psychosocial staffing at National Comprehensive Cancer Network member institutions: data from leading cancer centers. *Psychooncology.* 2016;25(2):164-169.
114. Garland SN, Xie SX, DuHamel K, et al. Acupuncture Versus Cognitive Behavioral Therapy for Insomnia in Cancer Survivors: A Randomized Clinical Trial. *J Natl Cancer Inst.* 2019;111(12):1323-1331.
115. Carroll BT, Kathol RG, Noyes R, Jr., Wald TG, Clamon GH. Screening for depression and anxiety in cancer patients using the Hospital Anxiety and Depression Scale. *General hospital psychiatry.* 1993;15(2):69-74.
116. Smith AB, Selby PJ, Velikova G, et al. Factor analysis of the Hospital Anxiety and Depression Scale from a large cancer population. *Psychology and psychotherapy.* 2002;75(Pt 2):165-176.
117. Lemay KR, Tulloch HE, Pipe AL, Reed JL. Establishing the Minimal Clinically Important Difference for the Hospital Anxiety and Depression Scale in Patients With Cardiovascular Disease. *Journal of cardiopulmonary rehabilitation and prevention.* 2019;39(6):E6-e11.
118. Mendoza TR, Wang XS, Cleeland CS, et al. The rapid assessment of fatigue severity in cancer patients: use of the Brief Fatigue Inventory. *Cancer.* 1999;85(5):1186-1196.
119. Morin CM, Belleville G, Belanger L, Ivers H. The Insomnia Severity Index: psychometric indicators to detect insomnia cases and evaluate treatment response. *Sleep.* 2011;34(5):601-608.
120. Savard MH, Savard J, Simard S, Ivers H. Empirical validation of the Insomnia Severity Index in cancer patients. *Psychooncology.* 2005;14(6):429-441.
121. Cleeland CS, Ryan KM. Pain assessment: global use of the Brief Pain Inventory. *Ann Acad Med Singapore.* 1994;23(2):129-138.
122. Bell ML, Dhillon HM, Bray VJ, Vardy JL. Important differences and meaningful changes for the Functional Assessment of Cancer Therapy-Cognitive Function (FACT-Cog). *Journal of Patient-Reported Outcomes.* 2018;2:48.
123. Hays RD, Bjorner JB, Revicki DA, Spritzer KL, Cella D. Development of physical and mental health summary scores from the patient-reported outcomes measurement information system (PROMIS) global items. *Quality of life research : an international journal of quality of life aspects of treatment, care and rehabilitation.* 2009;18(7):873-880.
124. Bauml J, Xie SX, Farrar JT, et al. Expectancy in real and sham electroacupuncture: does believing make it so? *Journal of the National Cancer Institute Monographs.* 2014;2014(50):302-307.
125. Keefe JR, Amsterdam J, Li QS, Soeller I, DeRubeis R, Mao JJ. Specific expectancies are associated with symptomatic outcomes and side effect burden in a trial of chamomile extract for generalized anxiety disorder. *Journal of psychiatric research.* 2017;84:90-97.
126. Beasley MJ, Ferguson-Jones EA, Macfarlane GJ. Treatment expectations but not preference affect outcome in a trial of CBT and exercise for pain. *Canadian journal of pain = Revue canadienne de la douleur.* 2017;1(1):161-170.

127. Mao JJ, Armstrong K, Farrar JT, Bowman MA. Acupuncture expectancy scale: development and preliminary validation in China. *Explore (NY).* 2007;3(4):372-377.
128. Mao JJ, Xie SX, Bowman MA. Uncovering the expectancy effect: the validation of the acupuncture expectancy scale. *Altern Ther Health Med.* 2011;16(6):22-27.
129. Liou KT, Trevino KM, Meghani SH, et al. Fear of analgesic side effects predicts preference for acupuncture: a cross-sectional study of cancer patients with pain in the USA. *Supportive care in cancer : official journal of the Multinational Association of Supportive Care in Cancer.* 2021;29(1):427-435.
130. Mas-Herrero E, Marco-Pallares J, Lorenzo-Seva U, Zatorre RJ, Rodriguez-Fornells A. Individual Differences in Music Reward Experiences. *Music Perception.* 2013;31(2):118-138.
131. Zhou Y, Lemmer G, Xu J, Rief W. Cross-Cultural Measurement Invariance of Scales Assessing Stigma and Attitude to Seeking Professional Psychological Help. *Frontiers in psychology.* 2019;10:1249.
132. Komiya N, Good GE, Sherrod NB. Emotional openness as a predictor of college students' attitudes toward seeking psychological help. *Journal of Counseling Psychology.* 2000;47(1):138-143.
133. Spitzer RL, Kroenke K, Williams JB, Löwe B. A brief measure for assessing generalized anxiety disorder: the GAD-7. *Archives of internal medicine.* 2006;166(10):1092-1097.
134. Bradt J, Burns DS, Creswell JW. Mixed methods research in music therapy research. *Journal of music therapy.* 2013;50(2):123-148.
135. Potvin N, Bradt J, Kesslick A. Expanding perspective on music therapy for symptom management in cancer care. *Journal of music therapy.* 2015;52(1):135-167.
136. Cox A, Lucas G, Marcu A, et al. Cancer Survivors' Experience With Telehealth: A Systematic Review and Thematic Synthesis. *Journal of medical Internet research.* 2017;19(1):e11.
137. Tango T. Power and sample size for the S:T repeated measures design combined with a linear mixed-effects model allowing for missing data. *Journal of biopharmaceutical statistics.* 2017;27(6):963-974.
138. Benjamini Y, Hochberg Y. Controlling the False Discovery Rate: A Practical and Powerful Approach to Multiple Testing. *Journal of the Royal Statistical Society Series B (Methodological).* 1995;57(1):289-300.
139. Benjamini Y, Yekutieli D. The Control of the False Discovery Rate in Multiple Testing under Dependency. *The Annals of Statistics.* 2001;29(4):1165-1188.
140. Kent DM, Rothwell PM, Ioannidis JP, Altman DG, Hayward RA. Assessing and reporting heterogeneity in treatment effects in clinical trials: a proposal. *Trials.* 2010;11:85.
141. Henderson NC, Louis TA, Wang C, Varadhan R. Bayesian analysis of heterogeneous treatment effects for patient-centered outcomes research. *Health services & outcomes research methodology.* 2016;16(4):213-233.
142. Wang C, Louis TA, Henderson NC, Weiss CO, Varadhan R. beanz: An R Package for Bayesian Analysis of Heterogeneous Treatment Effects with a Graphical User Interface. *2018.* 2018;85(7):31.
143. Imai K, Ratkovic M. Estimating treatment effect heterogeneity in randomized program evaluation. *Ann Appl Stat.* 2013;7(1):443-470.
144. Wager S, Athey S. Estimation and Inference of Heterogeneous Treatment Effects using Random Forests. *Journal of the American Statistical Association.* 2018;113(523):1228-1242.
145. Braun V, Clarke V. Using thematic analysis in psychology. *Qualitative Research in Psychology.* 2006;3(2):77-101.
146. Little RJ, D'Agostino R, Cohen ML, et al. The prevention and treatment of missing data in clinical trials. *N Engl J Med.* 2012;367(14):1355-1360.

147. Siddique J, Harel O, Crespi CM. Addressing Missing Data Mechanism Uncertainty using Multiple-Model Multiple Imputation: Application to a Longitudinal Clinical Trial. *The annals of applied statistics.* 2012;6(4):1814-1837.
148. Ibrahim JG, Molenberghs G. Missing data methods in longitudinal studies: a review. *Test (Madrid, Spain).* 2009;18(1):1-43.
149. Mao JJ, Liou K, Panageas K, et al. Effects of electroacupuncture and auricular acupuncture for chronic pain in cancer survivors: The PEACE randomized controlled trial. *Journal of Clinical Oncology.* 2020;38(15_suppl):12004-12004.
150. Liou KT, Baser R, Romero SAD, et al. Personalized electro-acupuncture versus auricular-acupuncture comparative effectiveness (PEACE): A protocol of a randomized controlled trial for chronic musculoskeletal pain in cancer survivors. *Medicine.* 2020;99(21):e20085.
151. Bowen GA. Naturalistic inquiry and the saturation concept: a research note. *Qualitative Research.* 2008;8(1):137-152.
152. Guest G, Bunce A, Johnson L. How many interviews are enough? An experiment with data saturation and variability. *Field Method.* 2006;18:59-82.
153. Trevino KM, Raghunathan N, Latte-Naor S, et al. Rapid deployment of virtual mind-body interventions during the COVID-19 outbreak: feasibility, acceptability, and implications for future care. *Supportive care in cancer : official journal of the Multinational Association of Supportive Care in Cancer.* 2020.

18.0 APPENDICES

Appendix A: Music Therapy Protocol

Appendix B: Cognitive Behavioral Therapy Protocol

Appendix C: Semi-Structured Interview Guide

Appendix D: Study Assessments

Appendix E: Recruitment Letter

Appendix EE: Recruitment Letter Men

Appendix EEE: Recruitment Letter Symptom Focused

Appendix F: Medication Diary

Appendix G: Additional Study Assessments

Appendix H: Music Therapy Worksheets

Appendix I: Cognitive Behavioral Therapy Worksheets

Appendix J: Recruitment Brochure

Appendix JJ: Recruitment Brochure Telehealth

Appendix JJJ: Recruitment Brochure Anxiety

Appendix JJJJ: Brochure Hands Greyscale

Appendix JJJJJ: Brochure Hands Color

Appendix K: No Contact Letter

19.0 MSK MULTICENTER TRIAL ADDENDUM

Not Applicable
